# The cellular basis of feeding-dependent body size plasticity in sea anemones

**DOI:** 10.1242/dev.202926

**Published:** 2024-07-09

**Authors:** Kathrin Garschall, Eudald Pascual-Carreras, Belén García-Pascual, Daria Filimonova, Annika Guse, Iain G. Johnston, Patrick R. H. Steinmetz

**Affiliations:** ^1^ Michael Sars Centre, University of Bergen, Thormøhlensgt. 55, N-5008 Bergen, Norway; ^2^ Department for Mathematics, University of Bergen, Allégaten 41, N-5007 Bergen, Norway; ^3^ Centre for Organismal Studies, Heidelberg University, Im Neuenheimer Feld 230, D-69120 Heidelberg, Germany; ^4^ Computational Biology Unit, Department of Informatics, University of Bergen, Thormøhlensgt. 55, N-5008 Bergen, Norway

**Keywords:** Sea anemone, Plasticity, Body size, Proliferation, Quiescence, Feeding, Starvation, *Nematostella*, Aiptasia

## Abstract

Many animals share a lifelong capacity to adapt their growth rates and body sizes to changing environmental food supplies. However, the cellular and molecular basis underlying this plasticity remains only poorly understood. We therefore studied how the sea anemones *Nematostella vectensis* and Aiptasia (*Exaiptasia pallida*) respond to feeding and starvation. Combining quantifications of body size and cell numbers with mathematical modelling, we observed that growth and shrinkage rates in *Nematostella* are exponential, stereotypic and accompanied by dramatic changes in cell numbers. Notably, shrinkage rates, but not growth rates, are independent of body size. In the facultatively symbiotic Aiptasia, we show that growth and cell proliferation rates are dependent on the symbiotic state. On a cellular level, we found that >7% of all cells in *Nematostella* juveniles reversibly shift between S/G_2_/M and G_1_/G_0_ cell cycle phases when fed or starved, respectively. Furthermore, we demonstrate that polyp growth and cell proliferation are dependent on TOR signalling during feeding. Altogether, we provide a benchmark and resource for further investigating the nutritional regulation of body plasticity on multiple scales using the genetic toolkit available for *Nematostella*.

## Teaser

**Summary:** Feeding and starvation in sea anemones induce growth and shrinkage, cell size changes and dynamic cell proliferation changes that support a nutritional, TOR-dependent control of organismal size and cell cycle progression.

## INTRODUCTION

Indeterminate growth describes the potential of organisms to grow throughout life. It is found among many bilaterian (e.g. fish, crustaceans) and non-bilaterian (e.g. cnidarians) phyla and is likely ancestral to animals (Hariharan et al., 2015; Sebens, 1987). This lifelong growth plasticity often correlates with high regenerative capacities and a high resilience to starvation stress (Hariharan et al., 2015; McCue, 2010). In oligotrophic environments, it allows for whole-body shrinkage as an extreme starvation response found commonly in ctenophores (Jaspers et al., 2015), cnidarians (Lilley et al., 2014), acoels (De Mulder et al., 2009; Nimeth et al., 2004) and planarians (Baguñà et al., 1990). Whole-body shrinkage is also found more sporadically in some protostomes [e.g. gastropods (Böer et al., 2007)] and vertebrates [e.g. lampreys (Olesen Larsen, 1962) and eels (Boëtius and Boëtius, 1985)].

Cnidarians (e.g. sea anemones, jellyfish, *Hydra*) generally exhibit high body plasticity and indeterminate growth (Sebens, 1987; Technau et al., 2015). Among cnidarians, feeding-dependent growth and starvation-induced whole-body shrinkage have been documented in scyphozoans (‘true jellyfish’) (Frandsen and Riisgård, 1997; Hamner and Jenssen, 1974; Lilley et al., 2014), hydrozoans (e.g. *Hydra*, *Clytia*) (Loomis, 1953; Matsakis, 1993; Muscatine and Lenhoff, 1965; Otto and Campbell, 1977) and anthozoans (sea anemones and corals) (Chomsky et al., 2004; Sebens, 1980).

Currently, the cellular and molecular processes underlying the feeding-dependent control of animal growth rates remain largely enigmatic (Vogt, 2012). This is mainly due to the evolutionarily derived genetic pre-determination of body sizes in most genetic research organisms (e.g. mammals, insects, nematodes), leading to an uncoupling of feeding and adult growth rates (Hariharan et al., 2015). How feeding regulates post-larval body growth on a cellular level has been studied mainly in the planarian *Schmidtea mediterranea* (Baguñà et al., 1990; González-Estévez et al., 2012; Newmark and Alvarado, 2000) and in *Hydra spp.* (Bosch and David, 1984; Buzgariu et al., 2014). Despite their phylogenetic unrelatedness, adult stem cells in both *Hydra* and planarians sustain a high overall cell turnover, and body size is controlled by balancing cell death and cell proliferation (Baguñà and Romero, 1981; Baguñà et al., 1990; Gambino et al., 2024; Otto and Campbell, 1977). Feeding induces a burst of cell proliferation that quickly declines to stable baseline levels during starvation (Baguñà and Romero, 1981; Bosch and David, 1984; Böttger and Alexandrova, 2007; González-Estévez et al., 2012). Cell cycles of *Hydra* stem cells are unusual, with a nearly absent G_1_ phase and a long G_2_ phase (Campbell and David, 1974; David and Campbell, 1972; Holstein et al., 1991). Starvation extends (by 1.5- to 2-fold) or arrests the *Hydra* cell cycle in some cell types but does not alter proliferation rates on the whole organism level (Buzgariu, 2014; Bosch and David, 1984). Planarian cell cycle phases appear to be more canonical but, as in *Hydra*, a slow-down or arrest of the cell cycle during starvation may be cell type-dependent, with little reduction in proliferation overall (Kang and Alvarado, 2009; Molinaro et al., 2021; Newmark and Alvarado, 2000).

Nutritional control of animal cell growth and cell cycle progression often involves signalling via the Target of Rapamycin (TOR) pathway. This highly intricate pathway is conserved between animals and yeast and senses intracellular nutrient levels to mainly control the biosynthesis of proteins, nucleotides and lipids (Cargnello et al., 2015; Laplante and Sabatini, 2012). In fed planarians, TOR kinase knockdown using RNAi leads to organismal growth arrest by reduced proliferation and increased cell death in neoblasts (Peiris et al., 2012; Tu et al., 2012). In *Hydra*, inhibition of TOR signalling by Rapamycin leads to increased autophagy but the role of TOR in body size and cell proliferation remains unexplored (Chera et al., 2009). Signalling via TOR kinase has recently been shown to also regulate nutrient sensing during the cnidarian-dinoflagellate symbiosis in *Exaiptasia diaphana* (Aiptasia; Voss et al., 2023). In *Nematostella* primary polyps, TOR signalling is essential for the induction of tentacle growth and global cell proliferation after the initiation of feeding (Ikmi et al., 2020).

Currently, the universality of strategies observed in planarians and *Hydra* to nutritionally regulate animal growth are difficult to assess given the limited sampling of taxa. Here, we reveal the quantitative organismal and cellular dynamics of indeterminate growth in two sea anemones: *Nematostella vectensis* and Aiptasia, the latter being facultatively associated with symbiotic, photosynthetic dinoflagellates (Davy et al., 2012; Rädecker et al., 2018).

**Fig. 1.**
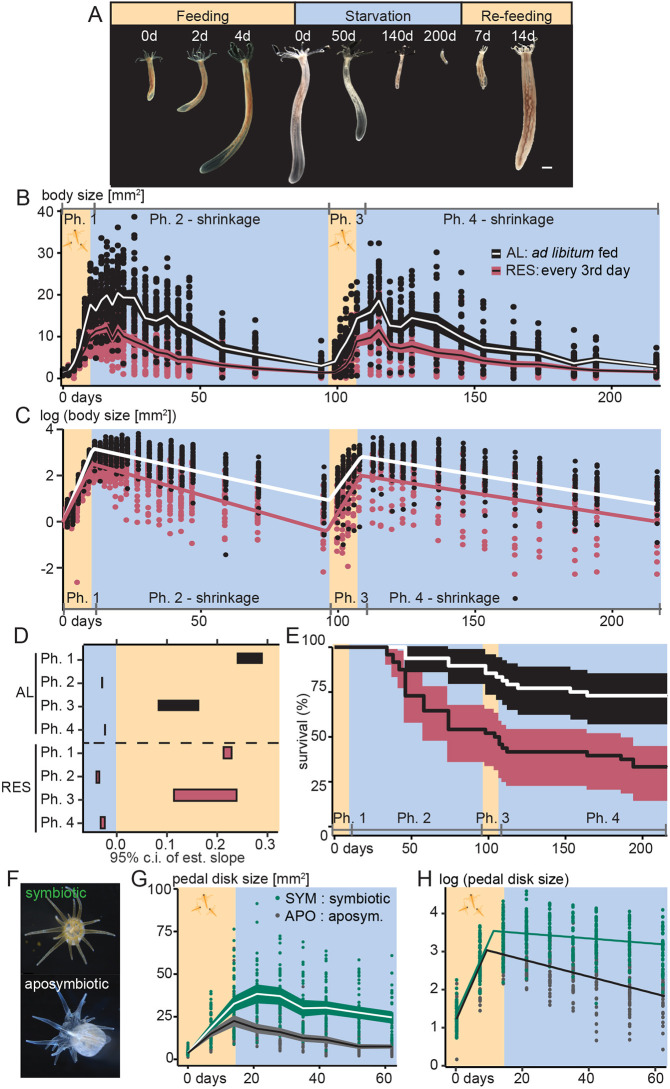
**Feeding-dependent body size plasticity in *Nematostella* and Aiptasia juveniles.** (A,F) Representative images of *Nematostella* juveniles (A) during starvation and re-feeding, and of symbiotic and aposymbiotic Aiptasia strain CC7 (F). (B-D) Comparison of *Nematostella* body size changes over two 10-day-feeding and 90-day-starvation cycles under *ad libitum* (AL) or restricted (RES; every third day) feeding regimes. (B) Body area measurements represented as individual values (dots) and mean values (lines) with a 95% confidence interval (c.i.) plotted and connected across different time points. At t_0_: *n*=96 individuals/condition in one experiment. (C) Representation of log-transformed values of body area with traces (lines) to illustrate mean behaviour of the four-phase model favoured by model selection (Fig. S1; supplementary Materials and Methods) under *ad libitum* (white line) or restricted (red line) feeding regimes. (D) Growth/shrinkage rate estimates from the slopes (95% confidence intervals) of multi-phase linear models on log-transformed body size values (see panel C; see also Fig. S1 and Table S1A). Note that estimated slopes directly correspond to growth/shrinkage rates. Colour code as in C. (E) Kaplan–Meier survivorship plot. Cox proportional hazard showed significantly reduced survival in polyps fed only every third day during feeding phases [Hazard Ratio=3.61, 95% confidence interval (1.89, 6.90), *P*<0.001]. Colour code as in C. (G) Aiptasia pedal disk area measured during 14 days of feeding and 56 days of starvation; At t_0_: *n*=36 individuals/condition in one experiment. (H) Log-transformed values of Aiptasia pedal disk area (G) with illustration of mean behaviour of two-phase model favoured by model selection (Fig. S5A-F). Ph., phase. Scale bar: 1 mm (A).

In *Nematostella*, feeding affects body size (Fig. 1A) (Carvalho et al., 2023 preprint; Hand and Uhlinger, 1992), and induction of tentacle growth depends on a global feeding-dependent proliferation (Ikmi et al., 2020). During starvation, *Nematostella* polyps shrink (Fig. 1A), reduce proliferation, retain characteristic locomotory behaviour and proportionally rescale the number of GLWamide-expressing neurons (Havrilak et al., 2021; Passamaneck and Martindale, 2012). In Aiptasia, growth is dependent on feeding, but it is unclear to what degree symbionts provide benefits to support growth or buffer starvation responses (Clayton and Lasker, 1985; Leal et al., 2012; Tivey et al., 2020). Until now, the quantitative dynamics of feeding- or starvation-induced changes on organismal, cellular and molecular levels have remained poorly understood in any animal, except in *Hydra* and planarians.

## RESULTS

### Body size in *Nematostella* is plastic and dependent on food supply

We tested how different feeding regimes affected growth or shrinkage in *Nematostella* by measuring polyp body column area (i.e. excluding tentacles, pharynx and mesenteries) as an approximation for body size (see Materials and Methods) (Reitzel et al., 2013). We compared two consecutive cycles of 10 days of *ad libitum* (AL) or restricted (every 3rd-day; RES) feeding followed by 90 days of starvation and observed that animals grew and shrank exponentially at feeding-dependent rates (Fig. 1B-D). To better understand the dynamics and characterise different phases of growth and shrinkage, we identified the change points in log-transformed body size and fitted linear models for the resulting intervals (Fig. 1C; Fig. S1A-R). The changes in body size were best described by a four-phase model with two growth (Fig. 1C; Fig. S1D,F,N,P; phases 1 and 3) and two shrinkage phases (Fig. 1C; Fig. S1D,F,N,P; phases 2 and 4). These largely overlapped with feeding and starvation phases; however, growth phases extended for some days into the starvation interval during all feeding regimes (Fig. 1C; Table S1A,B).

During the first growth phase (phase 1), we found major feeding-dependent differences between growth rates of AL (∼13-fold increase, doubling time T_D_:2.2-2.6 days) and RES (∼9-fold increase, T_D_:2.9-3.1 days) feeding (Fig. 1B-D; Fig. S2D; Table S1A). In the re-growth phase after starvation (phase 3), polyps under both feeding conditions generally grew slower than during phase 1 (Fig. 1B-D). Surprisingly, polyps under RES feeding tended to grow faster (T_D_:2.3-3.9 days) than AL-fed animals (T_D_:3.4-5.6 days) during phase 3 (Fig. 1D; Fig. S2D; Table S1A).

Shrinkage rates strongly differed between feeding regimes during the starvation phases 2 and 4 (Fig. 1D; Fig. S3K; Table S1A). During phase 2, AL-fed animals halved their size roughly every 4 weeks (halving time T_1/2_:26.5-31.3 days; Table S1A), whereas polyps with RES feeding shrank much quicker (T_1/2_:20.0-23.8 days; Table S1A). During phase 4, the difference between feeding regimes was less pronounced [T_1/2_(AL):33.8-41.5 days; T_1/2_(RES):27.6-40.8 days; Table S1A]. The generally lower shrinkage rates in both AL/RES regimes during phase 3 pointed to potentially increased starvation tolerance compared with phase 1. Comparing survival rates (Fig. 1E), we found that lethality during shrinkage phase 2 was higher under RES than AL. During phase 4, however, lethality was similar between feeding regimes and generally lower than in phase 2, likely resulting from selection for polyps with higher starvation resistance during phase 2. Interestingly, lethality also increased during re-feeding under both AL and RES (Fig. 1E; phase 3), suggesting that the transition from long-term starvation to re-feeding is accompanied by considerable metabolic stress.

### Growth and shrinkage rates are stereotypic and reproducible, but only shrinkage rates are independent of body size

**Fig. 2.**
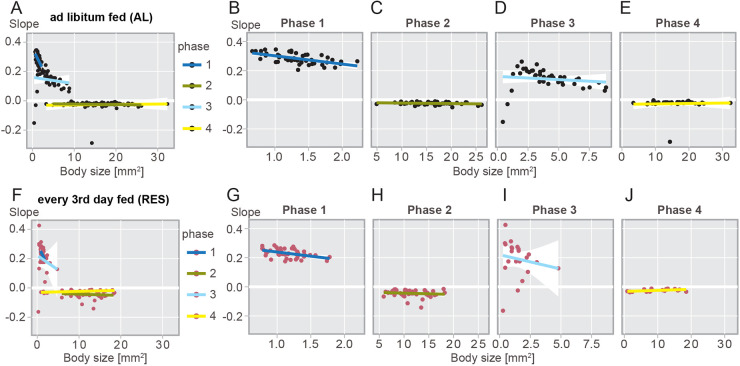
**Correlation between individual body size and growth or shrinkage rates.** (A-J) Slopes of *ad libitum* (A-E) or restricted (F-J) fed individuals represented with all phases combined (A,F) or as separated phases (B-E,G-J). Coloured lines indicate linear regressions per phase as colour-coded in A or F. White overlays represent a 95% confidence interval. Note that a horizontal regression line represents total independence of rates from polyp size.

Curious to study if growth or starvation rates were affected by body size, we tested whether, within a phase, the linear regression slopes of individuals were dependent on polyp starting sizes (Fig. 2A-J). As previously indicated (Fig. 1D), slope values were generally higher during growth phases (Fig. 2A,F; blue lines) than during shrinkage phases (Fig. 2A,F; yellow/green lines). If growth or shrinkage rates are independent from the starting body size within a phase, we would expect similar individual slopes across all sizes (‘horizontal’ distribution in Fig. 2). Indeed, we found that shrinkage rates during phases 2 and 4 are independent of polyp sizes for both feeding conditions (Fig. 2A,C,E,H,J; Table S1C). During growth phase 1, growth rates of smaller polyps were significantly higher than of larger polyps, with a stronger effect in AL than RES (Fig. 2A,F,B,G; Table S1C). During growth phase 3, the graph suggested a similar dependence, which was however not statistically significant, likely due to the large variation in slopes, especially among small polyps (Fig. 2D,I; Table S1C). Overall, 88% of the variance in slopes of the entire dataset could be explained by starting size, phase and feeding condition (Table S1C).

Researching the nutritional regulation of growth requires that growth rates are reproducible and stereotypic between independent but equivalent experiments (Fig. S2A-D). We compared growth rates between experiments and found that simple, single-phase linear regression models were a good fit to the data. Interested to study differences in the growth response between experiments, we tested whether the 95% confidence interval of the growth slopes intersected (Fig. S2D). We observed a large overlap between the slopes of AL growth phases when not preceded by starvation (Fig. S2D, 1 and 2). In addition, confidence intervals for growth rates also matched between all samples re-fed after starvation (Fig. S2D, 3, 5, 6 and 7). Together, and given the reasonably tight bounds on slope estimates reflected by these intervals, this result suggests a stereotypic and reproducible growth response to feeding dependent on starvation history and thus individual size.

We further asked whether polyp growth is isometric or allometric along the main axes and found that body length allometrically scaled to the square of body width. This result is consistent with anecdotal observations that juvenile polyp growth occurs predominantly in length (R^2^: 82%; Fig. 1A; Fig. S2E,F).

Body size changes were analysed using linear modelling with changepoints delimiting dynamic ‘phases’ with different slopes. We determined the best fitting model via Akaike Information Criterion (AIC) for up to eight different phases (Figs S1, S3A-J, S4A-D; Materials and Methods; supplementary Materials and Methods). Inference of the slopes in each phase and comparing their 95% confidence intervals showed that exponential shrinkage phases were similar within and between experiments (Fig. S3K). Together, our data suggested that *Nematostella* juveniles grow ∼6-12 times faster than they shrink. The general consistency of shrinkage rates between experiments, despite ∼5-fold variations in median starting body sizes (compare Fig. S3A and F), further confirmed that shrinkage rates are independent of starting size and that *Nematostella* responds stereotypically and highly reproducibly to starvation.

We then tested whether the growth and starvation responses were comparable between the estuarine *Nematostella* and the tropical marine sea anemone Aiptasia. Aiptasia is of particular interest to assess the effect of the presence or absence of photosynthetic symbionts on growth and shrinkage dynamics (Fig. 1F-H; Fig. S5A-G). We found that AL-fed Aiptasia showed similar pedal disk growth rates (Leal et al., 2012) between symbiotic (T_D_: 2.9-3.1 days) and aposymbiotic (T_D_: 3.6 days; Fig. 1G, Fig. S5A-G; Table S1A) polyps. During the first days after feeding, symbiotic polyps continued to grow, whereas aposymbiotic polyps started shrinking immediately and at ∼3-fold higher rates (T_1/2_: 24.3-31.4 days; Table S1A) than symbiotic polyps (T_1/2_: 55.8-3165.5 days) over 8 weeks (Fig. 1G,H). Generally, Aiptasia growth is ∼8- to 37-fold faster than shrinkage (Table S1A).

### Changes in cell size and cell numbers strongly correlate with nutrient supply in *Nematostella*

To test how cell numbers and/or cell sizes change during growth and shrinkage in *Nematostella*, we quantified body size, global cell numbers, median cell size and cell cycle distribution using imaging and flow cytometry approaches. The use of individual polyps allowed studying if parameters were correlated. Quantification of total cell numbers was both sensitive and scalable over a wide range (between 1:1 and 1:32 dilution factors; Fig. S6A). Adding a defined number of beads before maceration allowed identifying and normalising for the observed non-uniform cell loss during dissociation of starved samples (Fig. S6D; Materials and Methods).

**Fig. 3.**
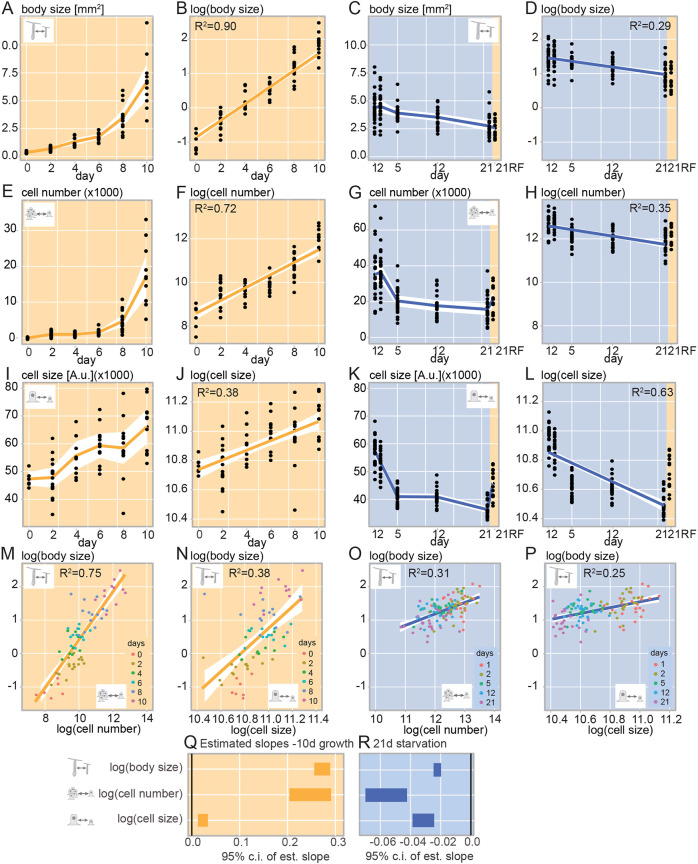
**Changes of body size, cell number and cell size during feeding or starvation.** (A-R) Changes of body size (A-D), cell number (E-H) and cell size (I-L) during 10 days *ad libitum* feeding (A,B,E,F,I,J,M,N,Q) or 21 days of starvation (C,D,G,H,K,L,O,P,R). Note that the re-feeding time point (21RF) in C,D,G,H,K,L represents samples taken 24 h after re-feeding at 20 days of starvation. (A,C,E,G,I,K) Representation of means and 95% confidence intervals (c.i.) connected across time points for individual animals. (B,D,F,H,J,L,N,P) Representation of linear models fit to log-transformed data. In D,H,L, the re-feeding (21RF) timepoint was excluded from model calculation (Fig. S4A-L; supplementary Materials and Methods). (M-P) Correlations between log-transformed values of body size and cell number (M,O) or cell size (N,P). (Q,R) Growth and shrinkage rate estimates (95% confidence interval) from linear regression analysis of log-transformed body size, cell number and cell size values during feeding (Q) and starvation (R). In all: *n*=24 individuals/time point in one experiment. A.u., arbitrary units; est., estimated.

Over 10 days of AL feeding, body size (Fig. 3A,B; T_D_: 2.3-2.5 days; Table S2C) and cell numbers (Fig. 3E,F; T_D_: 2.1-2.8 days; Table S2C) increased exponentially and log-transformed cell number correlated well with log-transformed body size changes (Fig. 3M; R^2^: 75%; Table S2D). Also, the rates in body size and cell number changes were similar (compare slope estimates in Fig. 3Q). Notably, median cell size continuously increased without peaking over 10 days of feeding (+40%; Fig. 3I,J; weak correlation to body size in Fig. 3N; R^2^: 38%), but at a much slower rate than body size (Fig. 3Q). A linear regression model to explain body size as a result of cell size (slope=0.858; s.d.=0.386) and cell number (slope=0.645; s.d.=0.061) provided a reasonable fit (R^2^: 77%; AIC: 95.80; Table S2E) and suggested that a power law relationship of the approximate form (body size)∼(cell size)^1^(cell number)^2/3^ links cell and body properties during growth (supplementary Materials and Methods, Geometry).

During starvation, body size (Fig. 3C,D) and cell numbers (Fig. 3G,H) increased slightly between days 1 and 2, followed by a major cell loss over the next 3 days (Δ_d5/d2_∼−44%) and towards day 21 (Δ_d21/d2_∼−57%) (Fig. 3G; Table S2B). We validated the flow cytometry results using Neubauer chamber counting on the same samples (Fig. S6B,C). The dramatic loss of cells between 2 and 21 days of starvation was particularly surprising as the median body size decreased by only ∼38% during the same interval (Fig. 3G; Table S2B). This was also reflected by the differences in slope estimates between cell numbers and body size changes (Fig. 3R). Consequently, log-transformed body size and log-transformed cell numbers are poorly correlated, suggesting a weaker relationship than during growth (R^2^: 31%; Fig. 3O; Table S2D). We further observed that cell numbers were highly plastic in response to feeding, as re-feeding of 20 days starved animals led to a strong increase of average cell numbers within 24 h (‘21RF’; Δ_d21/d21RF_+36%; Fig. 3G; Table S2B).

Also, median cell size, as measured by forward scatter area (FSC-A), decreased between day 1 and day 21 of starvation, although with an intermediate plateau (Δ_d21/d1_: −37%; Fig. 3K; Table S2B). We aimed to confirm this observation by using confocal microscopy to quantify and compare the area of single cells between 1 and 21 days of starvation (Fig. S6H-I). Despite the trend towards smaller sizes after 21 days of starvation, we did not see a significant difference in cell areas, likely owing to insufficient sample sizes (Fig. S6H-I; day 1: 50.2±2.6 µm^2^; day 42.4±3.3 µm^2^; pairwise *t*-test, *P*=0.174).

The decrease of median cell size could result from all cells generally becoming smaller, or from a change in the ratio between larger and smaller cells towards smaller size. We tested these alternatives by using size standards to define cell size bins in flow cytometry (Fig. S6E) and found that the fraction of smallest cells (2-4 µm diameter) increases over the course of starvation, whereas the proportion of all larger cell fractions (>4 µm) declined (Fig. S6F; see Fig. S10 for gating strategy). This change was reversible, as re-feeding animals starved for 20 days increased their median cell size within 24 h by increasing the fractions of larger cells (‘21RF’; Fig. S6F). Whether these shifts are because of the uptake of food particles in a subset of cells or a result of cell growth and cell cycle progression will need to be assessed.

Testing whether cell size and body size changes are correlated, we studied the relationship between log-transformed cell size and log-transformed body size. Similar to fed animals (Fig. 3N), we found that this relationship was too weak for estimating the effect in starved animals (R^2^: 25%; Fig. 3P; Table S2D).

### Feeding strongly induces a burst of cell proliferation in sea anemones

**Fig. 4.**
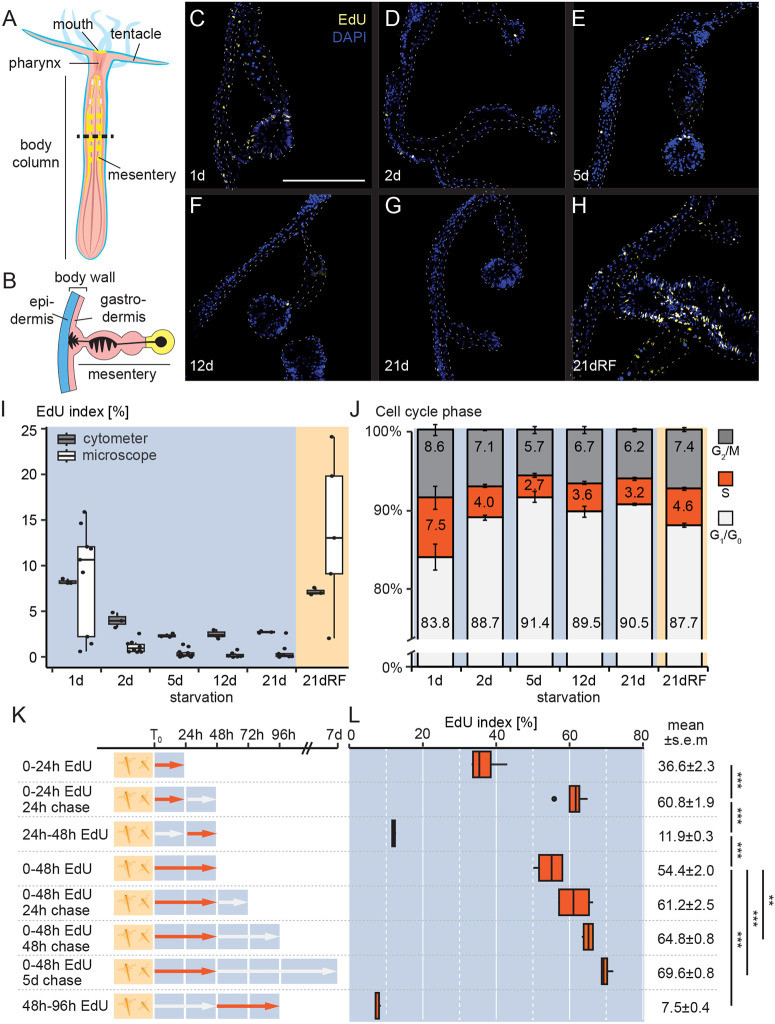
**Feeding and starvation strongly affect cell proliferation rates and cell cycle phase distributions.** (A,B) Schematic of a *Nematostella* polyp (A) and mesentery (B) indicating the approximate level (A; dashed line) and general morphology of mid-body cross sections analysed to determine the EdU index using confocal microscopy in C-I. (C-H) Representative confocal imaging stacks (maximum projections) of juvenile *Nematostella* polyps at mid-body cross section. EdU pulse labelling was started 60 min before fixation. (I) Quantification of the EdU index using imaging or flow cytometry (see also Table S3A,B). A two-way ANOVA revealed a non-significant effect of ‘assay’ [FC versus microscopy; *F*_(1, 60)_=0.05, *P*=0.817] and a marginally significant effect of ‘day’ [*F*_(5, 60)_=2.02, *P*=0.089] as well as a significant interaction between ‘assay’ and ‘day’ [*F*_(5, 60)_=2.56, *P*=0.037] on EdU levels. *n*(FC)=3 biological replicates with seven individuals per replicate; *n*(microscopy)=5-14 individuals. (J) Cell cycle phase distribution based on FxCycle violet DNA dye signal intensity. One-way ANOVA revealed a significant effect of ‘day’ on the EdU index [*F*_(5, 12)_=97.75, *P*<0.001] and on the proportion of S-phase cells [*F*_(5,12)_=7.60, *P*=0.002]. *n*=3 biological replicates with seven individuals per replicate. Polyps in C-J were starved for 1-21 days or sampled 24 h after re-feeding of 20-day-starved polyps (21dRF). (K,L) Experimental setup (K) and quantification of EdU index (L) in pulse-chase experiments. One-way ANOVA revealed a significant effect of experimental conditions (pulse-chase lengths and time points) on the EdU index [*F*_(7, 24)_=233.4, *P*<0.001]. Statistical significances of pairwise comparisons were calculated using Tukey's HSD post hoc test: ***P<0.001; **P<0.01. Duration of the chase had a strong effect on the EdU index and is consistent with ongoing cell proliferation (Table S3G). For all pulse-chase conditions: *n*=4 biological replicates. Orange arrows indicate periods of EdU pulse. White arrows indicate periods without EdU in the medium. Cell cycle composition and median fluorescence intensity shown in Fig. S7A-C. Boxes in I and L represent the first to the third interquartile ranges with median values (middle bars) and whiskers indicating the 1.5x IQR. Dots represent individual values (I) or outliers (L). Scale bar: 100 µm (C, for C-H).

Feeding-dependent changes in cell numbers suggested that food availability regulates cell division. We therefore estimated changes in cell proliferation rates in mid-body sections by using a confocal imaging approach (Fig. 4A-I), and in whole polyps by flow cytometry (FC; Fig. 4I). In both approaches, we detected and quantified S-phase cells labelled for 60 mins by the thymidine analogon 5-ethynyl-2′-deoxyuridine (EdU; Fig. 4C-I; Table S3A,B). Despite differences in methodology, we found that both methods indicated a strong decrease in the fraction of EdU^+^ cells (i.e. EdU index) between 1 and 5 days post-feeding (FC: 8.3±0.2% to 2.4±0.1%, Fig. 4I). Re-feeding after 20 days of starvation dramatically restored proliferation rates within 24 h (FC: 7.1±0.2% EdU^+^, Fig. 4I; Table S3A,B).

In Aiptasia, using a similar FC/EdU-labelling approach, we observed a significantly higher EdU index in symbiotic than in aposymbiotic polyps [two-way ANOVA, *F*_(1, 20)_=20.5, *P*=2.05E-04; Fig. S5H; Table S3J]. However, the EdU index differed significantly in both treatments between starvation time points [*F*_(4, 20)_=32.7, *P*=1.62E-08] and at similar rates [*F*_(4, 20)_=2.3, *P*=0.09] (Fig. S5H; Table S3J).

### Starvation induces G_1_/G_0_ accumulation in a large subset of proliferating cells

Although a short EdU pulse informs on the relative change in proliferation activity between time points, it only provides a temporal snapshot of S-phase cells, not all proliferating cells. To estimate global numbers of proliferating cells or cell cycle phase dynamics, we used FC-based DNA content measures to determine changes in the cell proportions of G_1_ or arrested G_0_ (2N DNA content), S (between 2N and 4N) and G_2_/M (4N) phases (Fig. 4J; Fig. S9). We found that, 24 h after feeding, at least 16.1%±2.1% cells in AL-fed *Nematostella* juveniles were proliferative cells (i.e. in either S or G_2_/M; Fig. 4J; Table S3B). The declining proportion of proliferative (S/G_2_/M-phase) cells over the first 21 days of starvation (d_1_: 16.1±2.1% to d_21_: 8.5±0.7%), mostly driven by S-phase decline, was accompanied by a complementary increase of G_1_/G_0_-phase cells (d_1_: 83.8±1.7% to d_21_: 90.5±0.1%) (Fig. 4J; Table S3B). Strikingly, re-feeding reversed this change in cell cycle proportions within 24 h (d_21RF_: 12.1±0.4 S/G_2_/M-phase; Fig. 4J). Our data thus suggest that starvation induces G_1_/G_0_ quiescence in ∼7% of cells that are competent to re-enter the cell cycle again upon re-feeding.

### Long-term EdU pulse substantiates high rates of cell addition at the onset of starvation

Using continuous EdU pulses, we aimed to estimate the contribution of cell divisions to the polyp body during the first 7 days after feeding (Fig. 4K; Table S3F; gating strategy: Fig. S11). We found that between 0 h and 24 h after feeding, 36.6±2.3% of cells incorporated EdU (73±0.9% in G_1_; Fig. 4K,L; Fig. S7A,C), whereas a 24 h/24 h pulse/chase experiment labelled 60.8±1.9% cells (84.9±0.5% in G_1_; Fig. 4K,L; Fig. S7A,C). This result indicates that cells labelled over the first 24 h continue dividing over the following 24 h, with an increasing fraction accumulating in G_1_ over the following 24 h, either due to terminal differentiation or G_1_/G_0_ quiescence. A continuous 24-48 h post-feeding (hpf) pulse resulted in a 11.9±0.3% EdU index (Fig. 4K,L), confirming that cell proliferation activity persisted, but at strongly decreased levels between 24 h and 48 h after feeding. The S/G_2_/M fraction increased between EdU^+^ cells labelled during 0-24 h (26.7±0.9%) or 24-48 h (36.7±1.1%) after feeding (Fig. S7C; Table S3G). The fraction of cells labelled on the second day after feeding is thus smaller, and cycle either slower and/or have a lower proportion of G_1_-differentiated cells in comparison with cells labelled within the first 24 h after feeding (Fig. 3L; Table S3G).

Given the dramatic drop in cell numbers between 2 and 5 days after feeding (Fig. 3G), we tested whether the cells dividing shortly after feeding were preferentially retained during this period of cell loss. We therefore analysed whether the proportion of cells labelled continuously by EdU between 0 and 48 h changed over the following days (Fig. 4K,L). We found that the EdU index significantly and continuously increased from 54.4±2.0% to 69.6± 0.8% after a 5 days long chase (Fig. 4K,L; Table S3G). This suggests that cells that proliferated within the first 48 hpf contributed substantially to the animal cell mass and were over-proportionally retained during subsequent shrinkage and cell loss. Labelling cells between 48 h and 96 h after feeding resulted in only 7.5%±0.4% EdU^+^ cells, with a relatively large proportion captured in S/G_2_/M cell cycle phases (36.7%±1.1%; Fig. S7A,C; Table S3F). This suggests the presence of a small population of slow-cycling cells that are only weakly or not dependent on nutritional input. Future studies (e.g. by single-cell RNA-sequencing) will further explore the nature of these cells and elucidate how starvation-induced cell loss and body shrinkage affects specific cell type populations.

### Re-feeding-induced polyp body growth, cell proliferation and median cell size increase are TOR signalling-dependent

**Fig. 5.**
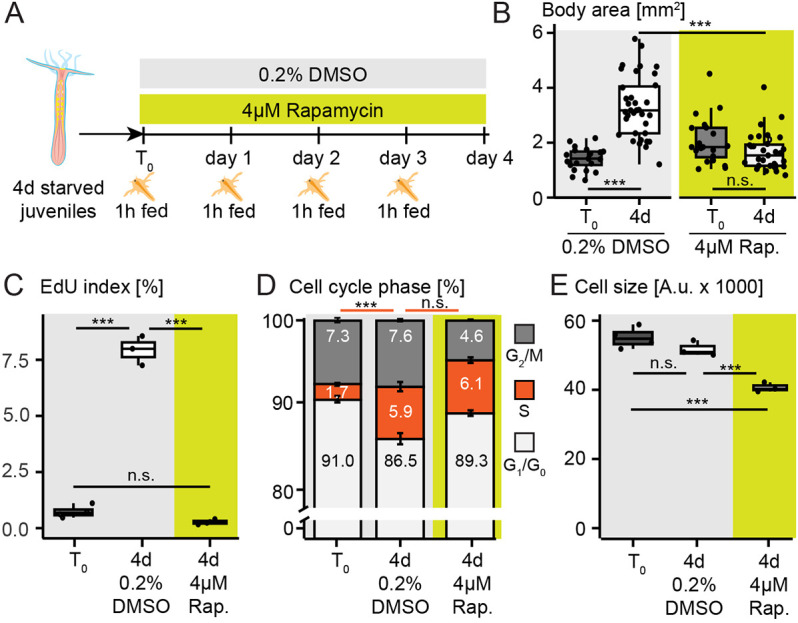
**Rapamycin blocks body growth and cell proliferation, and leads to mean cell size decrease in re-fed juveniles.** (A) Schematic overview of experimental setup. Note that polyps were without Rapamycin during the daily 1 h feeding period. (B-E) Quantification of body area with *N*=21-35 animals per timepoint (see Table S4A) (B), EdU index (C), cell cycle phase distribution based on DNA content (D) and mean cell size between T_0_, 0.2% DMSO controls and 4 µM Rapamycin using flow cytometry (E) (see Fig. S8 for details on TOR inhibition and cytometry and Table S4 for further details on statistics). In flow cytometry: *N*=3-4 biological replicates of pools of 7-10 animals. Significance codes for adjusted *P*-values: ***P<0.001. A.u., arbitrary units; d, days; n.s., non-significant; Rap., Rapamycin. Box plots show median values (middle bars) and the first to third interquartile ranges (box); whiskers indicate 1.5x IQR. Replicate sample values are plotted as dots.

Previous studies have shown that Tor complex 1 (TorC1) is required for cell proliferation and the induction of body and tentacle growth at the onset of feeding in primary polyps (Ikmi et al., 2020). We were therefore interested to test whether TOR signalling is mechanistically required during feeding-dependent growth, proliferation and median cell size increase in juvenile stages of *Nematostella*. TorC1 inhibition by a 4-day-long Rapamycin treatment (4 µM) under a daily feeding regime (Fig. 5A) led to a strong decrease in both the total abundance and the relative phosphorylation levels of the ribosomal protein S6 (Fig. S8A-D), previously used as TOR signalling readout in different sea anemones (Ikmi et al., 2020; Voss et al., 2023). In contrast to 0.2%DMSO controls that increased in size, animals incubated in 4 µM Rapamycin showed no significant difference in body size and EdU index while presenting significantly smaller median cell sizes between T0 and 4 days of treatment (Fig. 5B,C,E; Table S4)*.* Notably, however, the proportion of S-phase cells (based on DNA content) increased to levels similar to fed DMSO controls (Fig. 5D; Fig. S8E-G; Table S4G). These seemingly contradictory results suggest that Rapamycin treatment in re-fed juveniles induces a proportion of cells to enter and arrest during S-phase. This assumption was further supported by a decrease in the proportion of G_2_/M cells in Rapamycin-treated polyps in comparison with controls (Fig. 5D; Fig. S8G). Our data thus generally support a key role of TOR signalling in the control of feeding-dependent cell proliferation, body growth and cell growth.

## DISCUSSION

Sea anemones are well-known for their lifelong growth potential that allows them to adapt their sizes and growth rates to changing environmental conditions, e.g. food supply. In our study, we aimed to understand how the sea anemones *Nematostella* and Aiptasia respond to changes in food supply on cellular and organismal levels. For both species, we estimated body size changes by quantifying the body column area in *Nematostella* or pedal disk area in Aiptasia as proxies for the polyp body (Clayton and Lasker, 1985; Leal et al., 2012; Reitzel et al., 2013). Although we focused on analysing relative size changes, a more accurate measure of absolute changes in body size, mass, volume or surface area necessitates the development of volumetric imaging and quantification, e.g. by optical coherence microscopy, in sea anemones. In broad terms, *Nematostella* and aposymbiotic Aiptasia responded at 25°C with similar growth and shrinkage rates to feeding and starvation, respectively. In Aiptasia, the presence of symbionts delayed starvation-induced shrinkage and led to a consistently lower shrinkage rate (∼3-fold difference). These effects are likely due to the heterotrophic supply of nutrients from symbionts either by symbiont digestion and/or photosynthetic activity (Battey and Patton, 1984; Burriesci et al., 2012; Crossland et al., 1980; Revel et al., 2016).

We show that body growth and shrinkage rates in response to feeding and starvation in *Nematostella* are stereotypic, reproducible and asymmetric. The asymmetry is reflected by the observation that growth rates (or speed) in *Nematostella* (and also Aiptasia) are much higher than shrinkage rates. We also found that body size influences growth rates, but that shrinkage rates are independent of starting body sizes. The asymmetry between growth and shrinkage rates and the independence of shrinkage rates from body sizes are in strong contrast to recent data from planarians. Studies in *Schmidtea* have shown that growth and shrinkage occur at very similar rates, with both decreasing at increasing body size (Ko et al., 2024; Oviedo et al., 2003; Thommen et al., 2019).

In fed *Nematostella*, body growth and cell number increase are clearly positively correlated. Fed, larger polyps also contain cells of larger median cell sizes, likely resulting from a shifting ratio towards larger cells. In starved animals, median cell size and cell numbers increase 0-48 h (at 25°C) after feeding before decreasing from 48 h onwards.

So far, little is known about the changes in cell type composition during shrinkage except that the numbers of GLWamide neuropeptide-expressing neurons scales with body size during starvation (Havrilak et al., 2021). Revealing changes in the ratio and sizes of all cell types during juvenile growth – e.g. by a combination of flow cytometry-based cell size estimates and single-cell RNA-sequencing – will allow disentangling of the basis of median cell size changes during growth and shrinkage.

During starvation, the correlation between cell numbers and body size is weaker than during feeding conditions. This effect is likely due to the high level of cell loss, which is disproportionate to the observed body size reduction. This discrepancy could be explained by high cell losses in internal structures, differences in cell densities and/or a delay in extracellular matrix scaffold breakdown.

In *Hydra* and planarians, global cell loss during starvation was previously shown to mainly result from increased levels of apoptosis and changes in cell cycle progression (Baguñà and Romero, 1981; Bosch and David, 1984; Böttger and Alexandrova, 2007; González-Estévez et al., 2012). To better understand the basis of cell number changes in *Nematostella*, we estimated cell proliferation rates by quantifying the proportion of S-phase (EdU^+^) cells. We found that a continuous 24 h or 48 h-long EdU pulse after feeding results in high EdU labelling indices (>35% or >50%, respectively). This result shows that feeding induces high proliferation rates, in line with exponential cell number increases during growth. However, the stark difference of proliferative activity between the first and second day after feeding underlines that cell proliferation in *Nematostella* is highly dependent on food intake and that lack of continued supply leads to a prompt decline. In Aiptasia, EdU indices were higher in symbiotic than aposymbiotic animals despite a general reduction under starved conditions as previously suggested (Tivey et al., 2020, Gorman et al., 2022). Comparisons between *Nematostella* and Aiptasia show that EdU indices respond within similar ranges (∼1-7%) to food supply changes. In Aiptasia, however, the starvation-induced decrease was slower than in *Nematostella*. The similarities between *Nematostella* and Aiptasia indicate that the reversible control of cell proliferation by food supply is evolutionarily conserved between both and dates back at least to the last common ancestor of all sea anemones (Actinaria).

Using flow cytometry to estimate changes in cell cycle phases during starvation and re-feeding in *Nematostella*, we observe that ∼7% of cells can reversibly switch between S/G_2_/M and G_1_/G_0_ phases. Our data could be explained by an extreme and unlikely reduction of cell cycle progression speed >48 h after feeding. As *Nematostella* is starvation-resilient for many months, we favour the hypothesis that the observed cell proliferation dynamics are best explained by starvation-induced quiescence, where G_1_ arrested cells keep the potential to quickly re-enter the cell cycle again upon re-feeding. Our results also support that TOR signalling activity is required for organismal growth and cell proliferation after re-feeding in juvenile *Nematostella* polyps. Surprisingly, Rapamycin appears to arrest cells in S-phase, as indicated by low EdU incorporation but a relatively high proportion of S-phase cells determined by DNA content. Further work is necessary to test whether the effect of Rapamycin on cell proliferation occurs indirectly – e.g. as a consequence of histone depletion due to general inhibition of protein translation – rather than directly on controlling G_1_/S transition (Cargnello et al., 2015).

In most genetic research animals (e.g. mammals, flies, nematodes), post-larval quiescent stem or progenitor cells are rare (Cheung and Rando, 2013; Otsuki and Brand, 2020; Wong et al., 2023). In *Nematostella*, in contrast, the number of potentially quiescent cells in juvenile polyps is remarkably high (>7% of all cells). This difference in the proportion of proliferation-competent cells might be a key feature of indeterminately growing animals to ensure the feeding-dependent body growth plasticity. Future experiments will clarify whether cellular quiescence, a remarkable but poorly characterised cell state, is underlying nutritionally controlled growth plasticity in sea anemones and other indeterminately growing animals. Notably, starvation-induced quiescence is likely not predominant or sufficient to explain body size changes in planarians (Newmark and Alvarado, 2000) and *Hydra* (Bosch and David, 1984; Buzgariu et al., 2014).

We provide here a benchmark for feeding- and TOR-dependent changes in body size, cell size, cell numbers and proliferation rates in *Nematostella vectensis* and partly in Aiptasia. The ease of inducing quiescence and cell cycle re-entry in a large fraction of cells by starvation and re-feeding makes *Nematostella* one of the most tractable and accessible animal systems to study the nutritional control of the animal cell cycle. In combination with powerful genetic tools (e.g. transgenesis, CRISPR/Cas9-based tools) (Ikmi et al., 2014; Lebouvier et al., 2022; Paix et al., 2023; Renfer and Technau, 2017), our work puts *Nematostella* in the spotlight to continue studying the cellular and molecular basis of indeterminate animal growth control.

## MATERIALS AND METHODS

### Animal culture

All *Nematostella vectensis* polyps were wild-type laboratory strains (Hand and Uhlinger, 1992) kept at 16‰ salt concentration (*Nematostella* medium) in a 25°C incubator without lights. After animals reached primary polyp stage, they were fed daily with mashed *Artemia* nauplii for the first week and with live nauplii thereafter. During feeding phases, *Nematostella* medium was replaced daily. Symbiotic and aposymbiotic clonal lines of the *Exaiptasia diaphana* strain CC7 (Grawunder et al., 2015) were maintained at 25°C in 1.5 l plastic boxes with filtered sea water at a salinity of ±32‰. Symbiotic animals were kept in a 12 h light/12 h dark cycle, whereas aposymbiotic animals were maintained in darkness and regularly screened for the absence of symbionts using fluorescent microscopy. During feeding, all polyps were fed 5 days per week, with a full exchange of water twice a week, and a monthly scraping of the boxes. All experiments were performed on small polyps (∼3 mm pedal disk diameter) originating from pedal lacerates.

### Body size measurements in *Nematostella vectensis* and *Exaiptasia diaphana*

All experiments addressing growth and shrinkage were carried out in six-well plates with individually housed animals except for the ‘140d starvation’ experiment, during which pools of animals were kept in three replicate dishes. For shrinkage/starvation experiments, animals were fed until development of the second pair of full mesenteries (∼4-5 weeks after fertilization) as a morphological marker for juvenile polyps. Imaging was performed after relaxing animals using 0.1 M MgCl_2_ in *Nematostella* medium for 20 min using a Leica M165 FC microscope. After masking tentacles, we measured body pixel area in ImageJ/Fiji (Schindelin et al., 2015) by detecting contrasting objects and automatically outlining polyp shape using ‘analyse particles’. We then converted pixel area to mm^2^ based on pixel size of a 2 mm scale bar.

For body size measurements of Aiptasia, pedal lacerates of aposymbiotic and symbiotic animals were left to regenerate into small polyps and fed until reaching a minimum size of ∼3 mm diameter. Then 15-20 animals were selected per replicate dish (with three replicate dishes per symbiosis condition) and kept in a 12 h light/12 h dark cycle (photon flux density of about 40 µmol m^−2^ s^−1^) in an incubator at 25°C. During the first 14 days of the assay (feeding phase), animals were fed daily for 1 h with freshly hatched *Artemia* nauplii and washed 2-4 h later to remove food waste. During the starvation period, the assay dishes were cleaned using cotton swabs and sea water was replaced three times per week. Changes in body size were estimated by imaging and measuring Aiptasia pedal disk area after anaesthetization by incubation for a minimum of 15 min in 0.1 M MgCl_2_ in sea water (Angeli et al., 2016). Then, images were taken of pedal disks on an inverted microscope (Eclipse TE2000-S, Nikon), imported into the ImageJ/Fiji software and the pedal disk edge outlined by hand using a polygonal selection tool. The resulting area was recorded and converted to mm^2^.

### EdU labelling

Aiptasia or *Nematostella* polyps were relaxed for 15 min in 0.1 M MgCl_2_ in sea water or *Nematostella* medium and transferred to sea water or *Nematostella* medium containing 300 µM EdU (Invitrogen)/2% DMSO/0.1 M MgCl_2_. Animals were incubated at room temperature (RT) for 30 or 60 min for a short pulse and washed with 0.1 M MgCl_2_ in sea water or *Nematostella* medium. During long EdU pulse and chase experiments, animals were kept without MgCl_2_ in a 25°C incubator, and the EdU solution was replaced every 24 h. For flow cytometry analysis, animals were either dissociated using the acetic acid/methanol (ACME)- or Trypsin-based dissociation and storage protocols (see below). For microscopy sample preparation, the polyps were fixed in 3.7% formaldehyde/1× PBS overnight, washed three times in 1× PBS/0.5% Triton X-100 and stored in 100% methanol at −20°C.

For flow cytometry, rehydrated cells (see below) were permeabilized by incubation in 0.2% Triton X-100 in 1% bovine serum albumin (BSA)/PBS for 15 min at RT and washed once with PBS, followed by centrifugation at 800 ***g*** for 5 min at 4°C. The cell pellet was resuspended in 200 µl of freshly prepared Click-it reaction cocktail (C10337, Invitrogen) containing Alexa488 fluorophore azide (Invitrogen) and incubated for 30 min at RT in the dark. To reduce background, the cell suspension was washed twice with 1% BSA/PBS and stored at 4°C for flow cytometry analysis within 48 h. For whole mount samples, fixed polyps were washed twice in 1% BSA/1× PBS and permeabilized for 30 min at RT using 0.5% Triton X-100 in 1% BSA/1× PBS. After three washes with 1% BSA/1× PBS, the tissue was incubated in freshly prepared Click-it reaction cocktail with Alexa488 fluorophore azide at RT for 30 min in the dark, washed twice in 1% BSA/PBS and processed for cryo-sectioning.

### Cryo-sectioning

The sample polyps were infiltrated at 4°C with 25% sucrose/20% OCT (Tissue-Tek, Sakura)/1× PBS overnight and frozen in 80% OCT/1× PBS on a metal block cooled by liquid nitrogen. Using a Leica Cryostat, cross sections of 13 μm were collected from the polyp midbodies, transferred to SuperFrost adhesion slides (Thermo Fisher Scientific) and left to dry. After 24 h at RT, the tissue was re-fixed with 3.7% formaldehyde/PBS for 10 min and washed twice with 1× PBS before mounting in 80% glycerol for confocal microscopy.

### Tissue dissociation by ACME and flow cytometer sample preparation

Depending on the experiment, one to seven *Nematostella* polyps or three Aiptasia polyps constituted one biological sample and were transferred to tissue homogenization tubes (‘C-tubes’, 130-096-334, Miltenyi Biotec) after relaxation in 0.1 M MgCl_2_. ACME-based tissue dissociation was adapted from a previously described protocol (García-Castro et al., 2021) as follows. Polyps were transferred to 5 ml of freshly prepared ACME solution (15% methanol/10% glacial acetic acid/10% glycerol in Milli-Q water) and incubated for 1 h at RT with intermittent pulses of mechanical tissue disruption using the gentleMACS™ tissue homogenizer (Miltenyi Biotec). The resulting single cell suspension was washed twice using 10 ml of ice-cold 1% BSA/1× PBS and pelleted for 5 min at 800 ***g*** at 4°C. The cell pellet was gently resuspended in 1 ml of 1% BSA/1× PBS and filtered through a pre-wetted 50 µm CellTrics strainer (Sysmex). For storing samples up to a month at −20°C, cells were centrifuged (5 min at 800 ***g*** at 4°C) and taken up in 90% methanol. Before EdU labelling using a Click-It reaction and/or flow cytometry, cells were rehydrated by two washes with ice-cold 1% BSA/1× PBS (5 min at 400 ***g*** at 4°C).

### Flow cytometry – estimating cell numbers, cell size and cell cycle distributions

Cell suspensions (see above) were stained with 1 µg/ml FxCycle violet DNA dye (Invitrogen) within a day of the flow cytometry run. Flow cytometry was performed on a BD LSRFortessa (BD Life Sciences) and the resulting data were analysed using FlowJoV10.8 (BD Life Sciences). For analysis of *Nematostella* whole body cell suspensions (see Fig. S9-S12 for gating strategy), we first excluded debris based on size and granularity in the FSC-A/SSC-A gate, with sub-gates based on FSC-A/FSC-H parameters and FSC-A/SSC-W parameters to remove potential cell doublets and high complexity events. We then gated particles based on DNA dye intensity in width over area and plotted a histogram of DNA dye in area on the linear scale to visualize the characteristic DNA dye intensity peaks expected from cells between 2N and 4N. These pre-selected events were considered the pool of cells from which analyses of cell size (median FSC-A intensity in arbitrary units), cell cycle composition (subfractions based on DNA signal intensity) and cell counting were performed. It was also used to record the fraction of EdU^+^ cells, by subgating on EdU labelling fluorescence in reference to negative controls. In the long-term EdU pulse and chase experiments, the EdU^+^ population was further investigated using mean EdU fluorescence intensity (median value in arbitrary units) and cell cycle composition was established using DNA signal equivalent to the original gating without differentiation between S and G2M populations.

For a better interpretation of FSC-A recordings to investigate cell size changes during starvation, we used non-fluorescent polystyrene beads with known diameters between 2.0 μm and 15.0 μm (F13838, Invitrogen) and created a μm-sized reference of FSC-A values. Overlapping these size-bins with FSC-A values of cells in the cell cycle gate allowed us to assess how cell size fractions changed during starvation time points (see Figs S5E and S10 for gating strategy).

In cell counting experiments, 2×10^5^ 10 µm yellow-green FluoSpheres in PBS (F8836, Invitrogen) were added per animal before dissociation to quantify cell loss during dissociation. For estimating the number of cells per individual, 5×10^4^ 10 µm red FluoSpheres (F8834, Invitrogen) were added to the sample homogenate of a single polyp (see below). As these beads have a size comparable with cells (see Fig. S6G,H) and we know their numbers, we can estimate the total number of cells per sample by counting beads using the following calculation:

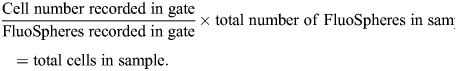
Proliferation assays in Aiptasia cell suspensions of both aposymbiotic and symbiotic animals were analysed with a slightly different strategy due to the autofluorescence of chlorophyll in algal symbionts (see Fig. S12). After excluding events based on size and granularity (FSC-A/SSC-A, FSC-A/FSC-H, FSC-A/SSC-W), we gated on particles without symbiont chlorophyll autofluorescence/SSC-A and focused our analysis on this fraction. DNA signal intensity was again plotted as a histogram and events that fell in the expected range for cell cycle (2N-4N) were considered the reference population from which the fraction of EdU^+^ cells were recorded by comparison with negative controls.

### Cell counting via Neubauer chamber

For validation of cell counts obtained by flow cytometry, the cell suspension was transferred to a Millicell^®^ Disposable Hemocytometer (MDH-2N1-50PK, Merck) counting chamber. According to manufacturer protocols, cells were manually counted and the mean number of cells per sample was calculated.

### Confocal imaging and EdU ratio determination using image analysis

Polyp mid-sections from EdU assays were imaged with a 20× oil-immersion lens using a Leica SP5 confocal microscope and standard PMT detectors. For validating cell size estimates, we stained the cell suspensions after flow cytometry with Alexa Fluor™ 488 Conjugate Concanavalin-A (2 µg/ml; C11252, Thermo Fisher Scientific) and imaged individual cells on an Olympus FLUOVIEW FV3000 confocal microscope (standard PMT detectors) with a 60× oil-immersion lens. Images were processed and cropped in ImageJ/Fiji (Schindelin et al., 2015). EdU quantification by imaging was based on stitched maximum projections of *z*-stacks from cryo-sections taken at mid-body level. Based on these images, the EdU index was determined using Imaris (Oxford Instruments) to infer the number of nuclei with EdU signal as a fraction of total nuclei.

### Trypsin/formaldehyde-based cell dissociation and fixation

A pool of 7-10 animals was dissociated based on previously published protocols (Buzgariu et al., 2022; Torres-Mendez et al., 2019), and the resulting cell suspension treated as a single biological replicate. Briefly, after relaxation with 0.1 M MgCl_2_, animals were washed with Ca^2+^- and Mg^2+^-free *Nematostella* medium (CMF/NM) and subsequently with CMF/NM containing 0.195% ethylenediaminetetraacetic acid (EDTA) (resulting in CMF/NM+E). After, animals were incubated for 5 min at 37°C with pre-heated CMF/NM+E containing 0.25% Trypsin (w/v). Homogenization was performed by pipetting and trypsinization was stopped by adding cold CMF/NM containing 1% BSA and 2.5% of fetal bovine serum. Cells were centrifuged for 5 min at 800 ***g*** at 4°C, resuspended in 1× PBS/1% BSA (w/v), filtered through a pre-wetted 50 µm CellTrics strainer (Sysmex) and fixed with 3.7% formaldehyde for 30 min at RT in the dark. The resulting cell suspension was washed twice with 1× PBS/1% BSA after centrifugation at 800 ***g*** at 4°C for 5 min, resuspended in 90% methanol/10% 1× PBS and stored at −20°C. Cells were rehydrated by two washes with ice-cold 1% BSA/PBS (5 min at 400 ***g*** at 4°C) and stained for flow cytometry.

### Immunofluorescence and S-phase detection on WM

Juvenile polyps were relaxed in 0.1 M MgCl_2_, then fixed in 3.7% formaldehyde/NM for 1 h at RT and dissected in the same solution in a Petri dish as previously described (Miramon-Puertolas and Steinmetz, 2023 preprint). Fixative was washed away thoroughly in 1× PBS/0.2% Tween20, followed by dehydration in a methanol series (20%, 50%, 100%) in 1× PBS/0.2% Tween20. Samples were washed in 100% methanol until pigment was completely removed from the tissue and stored in 100% methanol at −20°C. After progressive rehydration of the tissue in 1× PBS/0.2% Triton X-100, S-phase detection was performed using the Click-iT™ EdU Cell Proliferation Kit for Imaging (Thermo Fisher Scientific, C10337) following the manufacturer's protocol. After a 30 min incubation in the Click-it staining reaction, tissue pieces were washed in 1× PBS/0.2% Triton X-100, followed by a blocking step in 1× PBS/10% DMSO/5% normal goat serum (NGS)/0.2% Triton X-100 for 2 h at RT. The rabbit anti-p-RPS6 Ser235/236 primary antibody incubation (Cell Signaling Technology, 4858) was used at 1:50 dilution in 1× PBS/0.1% DMSO/5% NGS/0.2% Triton X-100 for 3 nights at 4°C. After six washes over 1 h of 1× PBS/0.2% Triton X-100 washes, the tissue was blocked in 1× PBS/5% NGS/0.2% Triton X-100 for 30 min at RT. Hoechst 33342 nuclear dye was used at 10 µg/ml (Life Technologies, H3570) and goat anti-rabbit-Alexa568 secondary antibody (LifeTech, A11011) was diluted to 1:500 in 1× PBS/5% NGS/0.2% Triton X-100 and incubated together overnight at 4°C. Finally, tissue pieces were washed thoroughly in 1× PBS/0.2% Triton X-100 and mounted in 80% glycerol.

### Western blotting

Protein was extracted from a pool of 50 juvenile polyps. After relaxation using 0.1 M MgCl_2_, animals were transferred to homogenization tubes (‘M-tubes’, Miltenyi Biotec, 130-093-236) containing RIPA buffer (150 mM NaCl, 50 mM Tris pH 8.0, 1% NP40, 0.5% DOC, 0.1% SDS) supplemented with cOmplete™ EDTA-free Protease Inhibitor Cocktail (Roche, 4693159001) and PhosphoStop inhibitor EDTA-free (Roche/Merck, 4906837001). Samples were incubated for 30 min on ice with intermittent pulses of mechanical tissue and cellular disruption using the pre-installed ‘protein_01_01’ programme on the gentleMACS™ tissue homogenizer (Miltenyi Biotec, 130-093-235). The resulting homogenate was centrifuged for 15 min at 4°C at 14,000 ***g*** and the supernatant transferred to a new tube and stored at −80°C. The protein concentration was quantified using the Bradford Assay Kit (Thermo Fisher Scientific, 23246) following the manufacturer's protocol. For SDS-PAGE sample preparation, 20 µg of protein was mixed 4:1 with 4× Laemmli sample buffer (0.1 M TrisHCl pH 6.8, 2% SDS, 20% Glycerol, 4% β-mercaptoethanol, 0.02% Bromophenol blue) and boiled for 5 min before loading. SDS-PAGE was performed using 7.5% Mini-PROTEAN^®^ TGX™ precast protein gels (Bio-Rad, 4561023) run in running buffer (25 mM TrisHCl, 192 mM Glycine, 0.1% SDS) at 100 V for ∼90 min. The 10-250 kDa PageRuler™ Plus pre-stained protein ladder (Thermo Fisher Scientific, 26619) was used as standard. Blotting was performed using Trans-Blot Turbo Mini 0.2 µm PVDF Transfer Pack (Bio-Rad, 1704156) on a Trans-Blot Turbo transfer system (Bio-Rad) with the mixed molecular weight programme. After protein transfer, membranes were washed with 1× PBS/0.1% Tween (PBT) several times and blocked with 5% milk powder in PBT (MPBT) at RT for 1 h. The blots were cut using the 35 kDa band as reference and incubated using the following primary antibodies in MPBT overnight at 4°C: rabbit anti-p-RPS6 Ser235/236 (1:5000; Cell Signaling Technology, 4858), rabbit anti-RPS6 (1:2000; Cell Signaling Technology, 2217T) and rabbit anti-Actin (1:2000; Sigma-Aldrich, A5060). The membranes were washed several times in PBT and incubated in goat anti-rabbit-HRP (1:10,000; Abcam, Ab9705) secondary antibody in MPBT at RT for 1 h. Membranes were then washed several times in TBT (20 mM TrisHCl, 150 mM NaCl, 0.1% Tween pH 7.6) and the signal was revealed using Clarify ECL substrate (Bio-Rad, 1705060) and imaged on a ChemiDoc XRS+ (Bio-Rad). Quantifications were performed with ImageJ software, measuring pixel intensity per loading condition and subtracting the background intensity. The pRPS6 and RPS6 protein signal was normalized with Actin signal.

### Rapamycin treatment

A previously published protocol for Rapamycin incubation (Ikmi et al., 2020) was adapted as follows: a 10 mM Rapamycin (Sigma-Aldrich, R8781) stock solution in 100% DMSO was diluted in *Nematostella* medium to a final concentration of 4 μM. Incubation in 4 µM Rapamycin or 0.2% DMSO (control) occurred at 25°C in the dark, and solutions were exchanged daily after 1 h of feeding. Rapamycin or DMSO treatment was carried out for 4 days.

### Data analysis and mathematical modelling

Data visualization and statistical analysis were performed using R (https://www.r-project.org/) with the libraries lmtest (Zeileis and Hothorn, 2002), ggplot2 (Wickham, 2016), gridExtra (https://rdrr.io/cran/gridExtra/) dplyr (https://dplyr.tidyverse.org/), tidyverse (Wickham et al., 2019), ggridges (https://wilkelab.org/ggridges/), survival (Grambsch and Therneau, 2000) and the Python libraries matplotlib (Hunter, 2007), Numpy (Harris et al., 2020), pandas (McKinney, 2010) and seaborn (Waskom, 2021). A detailed report on the models for analysing growth and shrinkage data can be found in the supplementary Materials and Methods (Statistical methods). In short, values for body size, cell number and cell size were natural logarithm transformed to better approximate homoscedastic, normally-distributed variables, and were used as input into linear regression models.

As we anticipated several phases in the body size response (especially during starvation), we determined the intervals for the linear regression by employing a multi-phased changepoint-model approach that optimised fit by maximising the log likelihood function (see supplementary Materials and Methods, Doubling times, half-lives and interval loss rates in starvation for further details). To estimate uncertainty in the parameter values, we employed bootstrap resampling (Berrar and Dubitzky, 2013). AIC values were calculated for each dataset to support model selection between different dynamic structures. Positive slope values obtained from linear regressions of natural logarithm transformed data could then be interpreted as exponential growth (expressed as doubling time, T_d_) and negative slope values as exponential decay/shrinkage rates (expressed as halving time, T_1/2_) with an overlap of the slopes (in the 95% confidence interval) considered a similar response. To address whether the individual growth or shrinkage rates were affected by the starting size, we analysed the correlation of growth or shrinkage rates per individual with body size at the start of each of the feeding/starvation phases by linear regression. A negative or positive slope of this regression line points to a dependence of the growth/shrinkage rate on the starting body size.

For our analysis of EdU experiments, we employed ANOVA to estimate the effects of assay parameters (factors) and conducted a Tukey's honest significant difference (HSD) test to evaluate the statistical significance of differences between experimental groups (see Tables S1-S4).

## Supplementary Material

10.1242/develop.202926_sup1 Supplementary information

Table S1A. Slope estimates from multi-phase linear models for *Nematostella* and Aiptasia body size changes **Table S1B.** Changepoints in multi-phased linear models for *Nematostella* and Aiptasia body size changes
**Table S1C.** Analysis of *Nematostella* growth / shrinkage rates in dependence of the individual starting size, phase (1-4) and feeding condition (RES vs AL)

Table S2A. Summary of body size, cell number, cell size values during *Nematostella* growth and shrinkage **Table S2B.** Changes in body size, cell number and cell size between timepoints
**Table S2C.** Linear regression analysis of individual bodysize, cell number and cell size
**Table S2D.** Linear model to estimate the effect of cell size or cell number on body size
**Table S2E.** Linear model to estimate the combined effect of cell size and cell number on body size
**Table S2F.**Pairwise comparisons (Tukey's HSD) between starvation timepoints based on an ANOVA to describe body size, cell number or cell size changes

Table S3A. Fraction of EdU positive cells as counted by images from confocal microscopy **Table S3B.** Flow cytometer analysis after 60min of EdU pulse - cell cycle phases defined as per DNA content
**Table S3C.** Comparison of the effect of the assay method (microscopy vs cytometry), day, and their interaction on the EdU index (ANOVA)
**Table S3D:** ANOVA for the effect of starvation day on the fraction of EdU positive cells and pairwise comparisons between days (Tukey's HSD)
**Table S3E.** ANOVA for the effect of starvation day on the fraction of EdU positive cells and pairwise comparisons between days (Tukey's HSD)
**Table S3F.** Flow cytometer analysis of long term EdU pulse (>24h) - cell cycle phases defined as per DNA content
**Table S3G.** ANOVA for the effect of experimental treatment on the fraction of EdU+ cells and pairwise comparisons between treatments (Tukey's HSD)
**Table S3H.** ANOVA for the effect of experimental treatment on the fraction of EdU+ S/G2M cells and pairwise comparisons between treatments (Tukey's HSD)
**Table S3I.** ANOVA for the effect of experimental treatment on the log (FITC-A) intensity as a quantification of EdU incorporation in EdU positive cells and pairwise comparisons between treatments (Tukey's HSD)
**Table S3J.** ANOVA for the effect of symbiotic state (symbiotic/aposymbiotic) on the fraction of EdU positive cells in Aiptasia and pairwise comparisons between treatments (Tukey's HSD)

Table S4A. Bodysize before and after 4 days of daily feeding under DMSO (0.2%) or Rapamycin (4μM) treatment **Table S4B.** EdU index before and after 4 days of daily feeding under DMSO (0.2%) or Rapamycin (4μM) treatment
**Table S4C.** Cell cycle composition before and after 4 days of daily feeding under DMSO (0.2%) or Rapamycin (4μM) treatment
**Table S4D.** Median FSC-A as a proxy for cell size before and after 4 days of daily feeding under DMSO (0.2%) or Rapamycin (4μM) treatment
**Table S4E.** ANOVA for the effect of day (day 0 vs day 4) and treatment (DMSO (0.2%) vs Rapamycin (4μM) on bodysize
**Table S4F.** ANOVA for the effect of DMSO (0.2%) or Rapamycin (4μM) treatment on the EdU index
**Table S4G.** ANOVA for the effect of DMSO (0.2%) or Rapamycin (4μM) treatment on the fraction of cells in S-phase
**Table S4H.** ANOVA for the effect of DMSO (0.2%) or Rapamycin (4μM) treatment on median FSC-A (as proxy for cell size)
