# Supplementary material for: The cellular basis of feeding-dependent body size plasticity in sea anemones
Source: Development. 2024 Jul 9;151(20):dev202926. doi: 10.1242/dev.202926 (PMC11267454; doi:10.1242/dev.202926)
Supplement: Supplementary information [file develop-151-202926-s1.pdf]

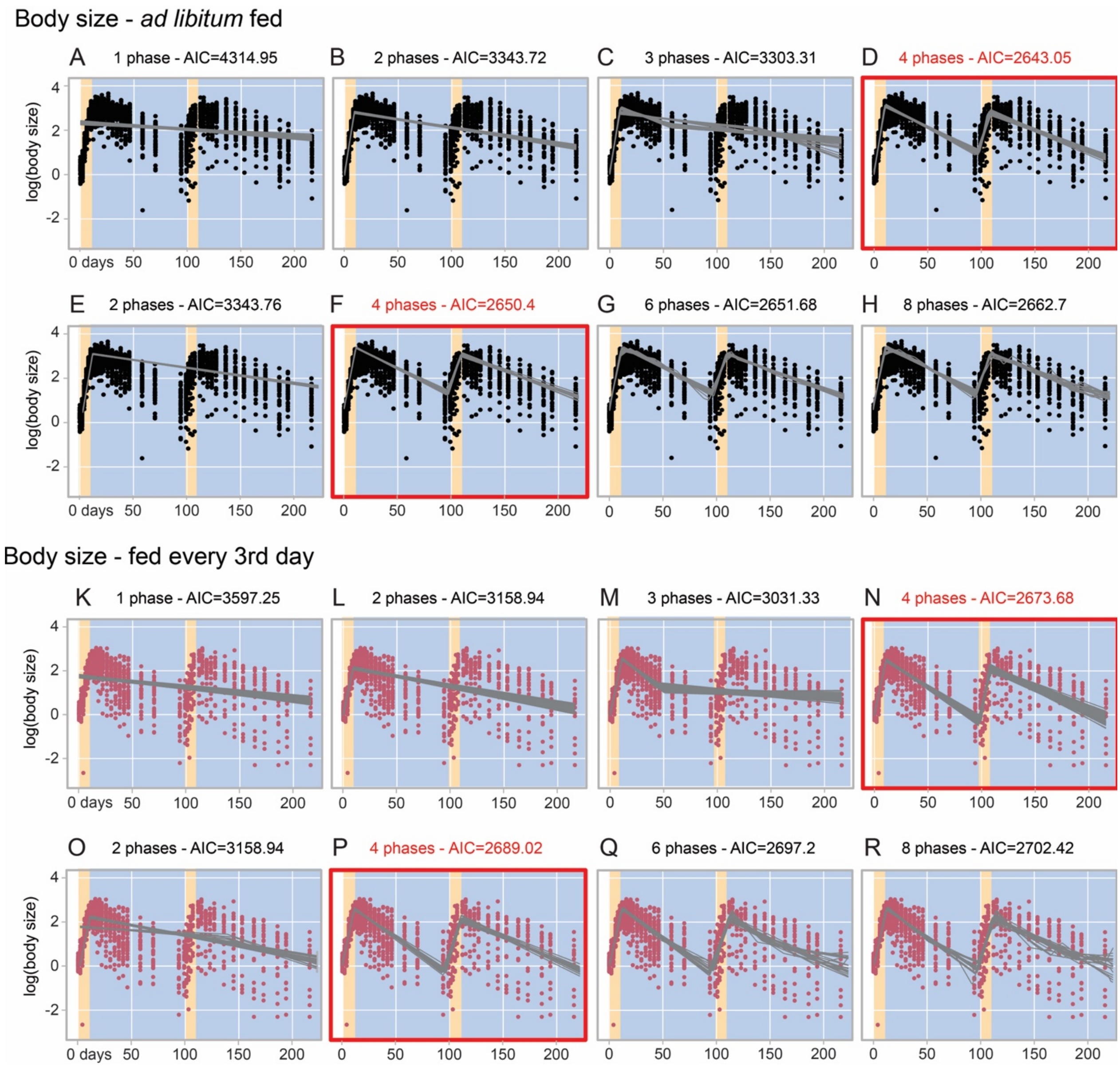

Fig. S1. Model selection to estimate growth and shrinkage phases during *ad libitum* or restricted (every 3rd day) feeding by maximizing log likelihood on log transformed body size data. Comparison of multi-phased linear regression models for log-transformed body size data assuming 1 to 4 phases (A-D, K-N) and a simulated annealing approach for 2-8 phases (E-H, O-R), using the Akaike Information Criterion (AIC). The best statistical fit (lowest AIC) for both feeding conditions was a 4-phase model parameterized using simulated annealing (red boxes). See phases in Fig. 1B, C. For details on the model parameters, see SI and Table S1A, B.

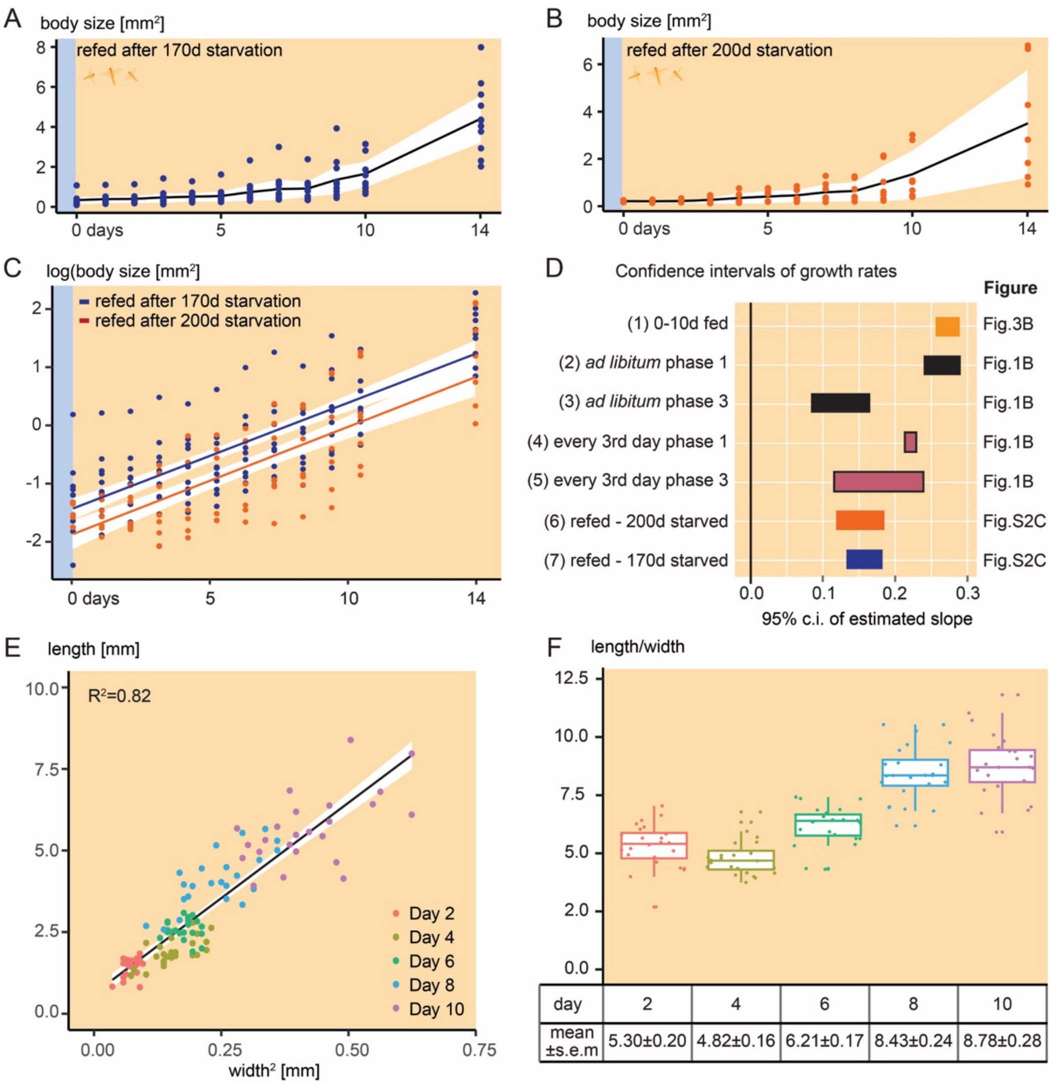

Fig. S2. Stereotypy of growth during feeding or re-feeding of *Nematostella* juveniles. (A-C) Body size (A,B) and log-transformed body size (C) of *Nematostella* juveniles starved for 170 (A) or 200 days (B) after 14 days of *ad libitum* feeding. In (A, B), means and 95% confidence intervals (c.i.) are connected across time points. At t_0: n= 12 individuals per time point in both experiments (A, B). (C) Representation of linear regression model on the log transformed values. (D) Representation of growth rate estimates from fitting linear models to log-transformed body size values. Note overlap of slopes in animals fed *ad libitum* without prior starvation (see (1)-(2)) and of slopes from experiments with prior starvation (see 'phase 3' and 'refed' in (3) and (5)-(7)). (E, F) Length scales with the squared width in polyps fed *ad libitum* over 10 days (see also SI: Geometry). Body area measurements of the same individuals as shown in Fig. 3A. n=24 individuals per time point. c.i.: confidence interval; s.e.m.: standard error of mean.

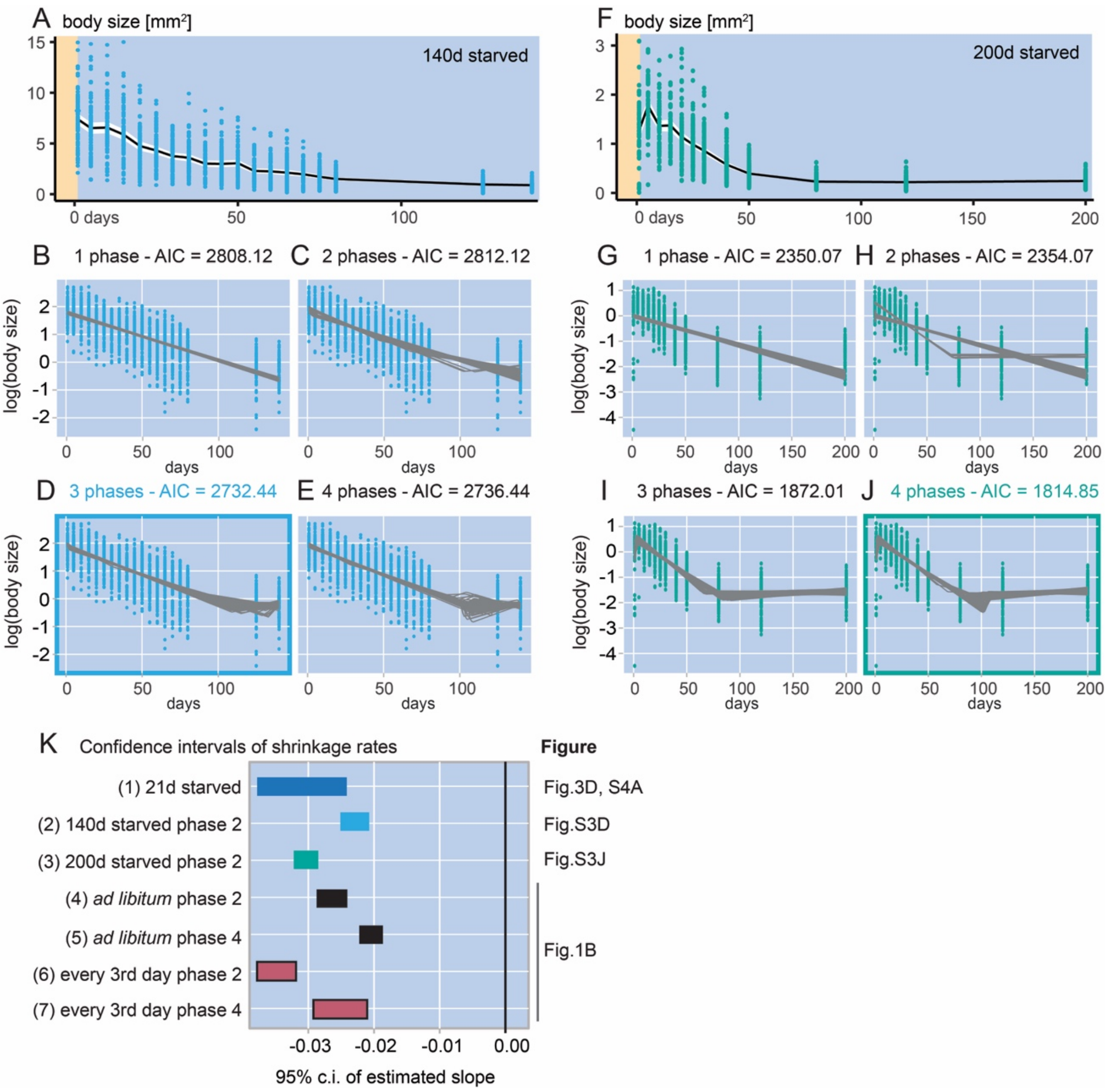

Fig. S3. Stereotypy of shrinkage during starvation in *Nematostella* juveniles. (A-J) Body size (A, F) and log-transformed body size (B-E, G-J) during 140 (A-E) or 200 (F-J) days of starvation. Black lines in (A, F) represent connected mean values and white overlay represents the 95% confidence interval per time point. At t_0: n=90 individuals (A) or n=between 58 and 136 individuals from a larger pool (F). (B-E, G-J) Representations of multi-phased linear regression models for log-transformed body size during 140 days of starvation (B-E) or 200 days of starvation (G-J) with 100 bootstraps visualized as grey lines. Lowest Akaike Information Criterion (AIC) indicates best fit models highlighted by boxes (D, J; see SI). (K) Comparison of estimated slopes (in 95% confidence intervals (c.i.)) for all experiments on *Nematostella* shrinkage. Note that all slopes are narrowly distributed between approx. -0.04 and -0.02.

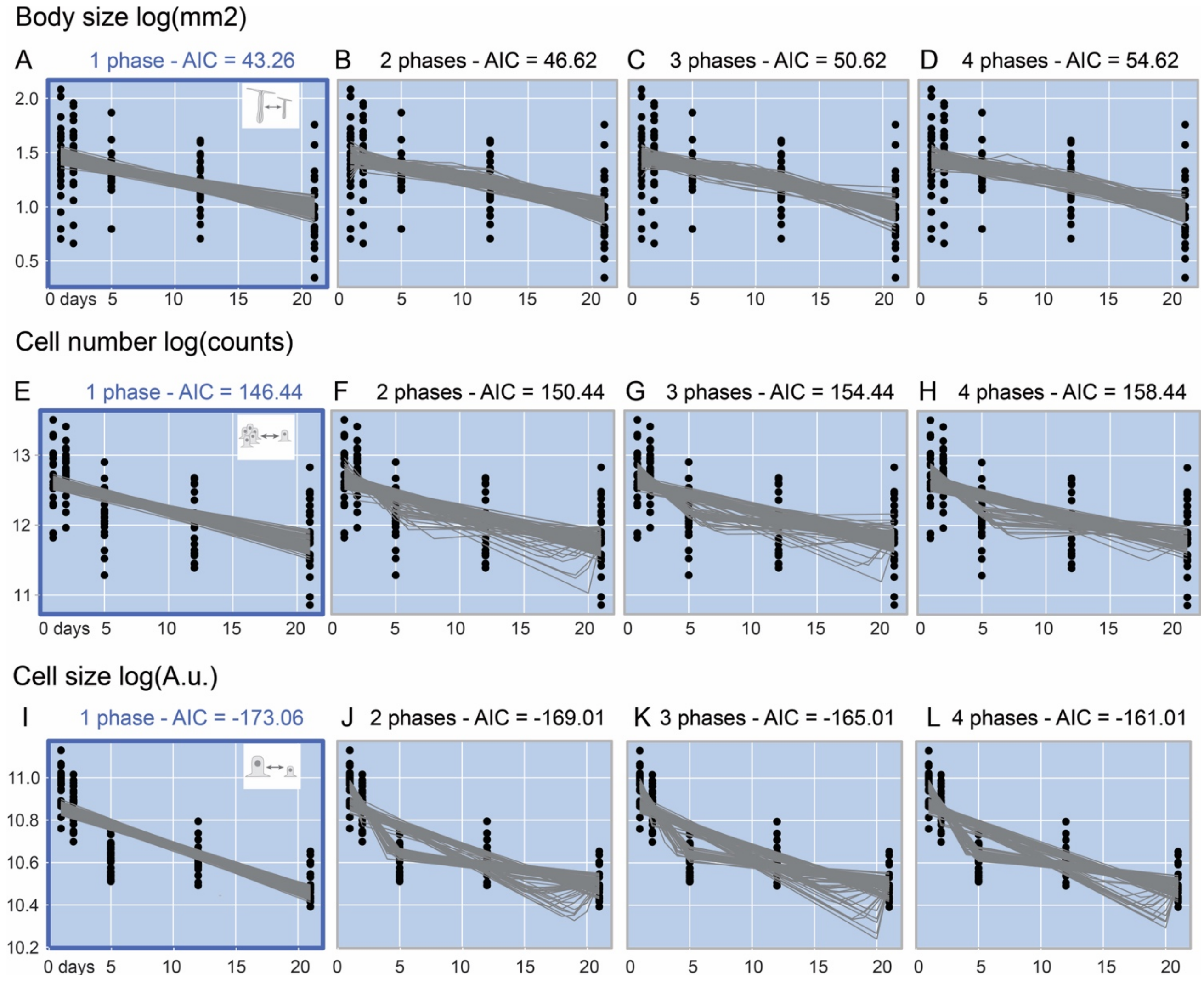

Fig. S4. Body size (A-D), cell number (E-H) and cell size (I-L) dynamics during 21 days of starvation in *Nematostella* juveniles. For 21 days starvation, multi-phased linear regression models were fitted to the log-transformed values of body size, cell number and cell size from individual polyps. See also Fig. 3D, H, L. 100 bootstraps (in grey lines) were plotted to test 1 to 4 models. Best fitting models highlighted by blue boxes as based on Akaike Information Criterion (AIC; see SI). A model with one shrinkage phase (A, E, I) described body size, cell number and cell size changes best over 21 days of starvation.

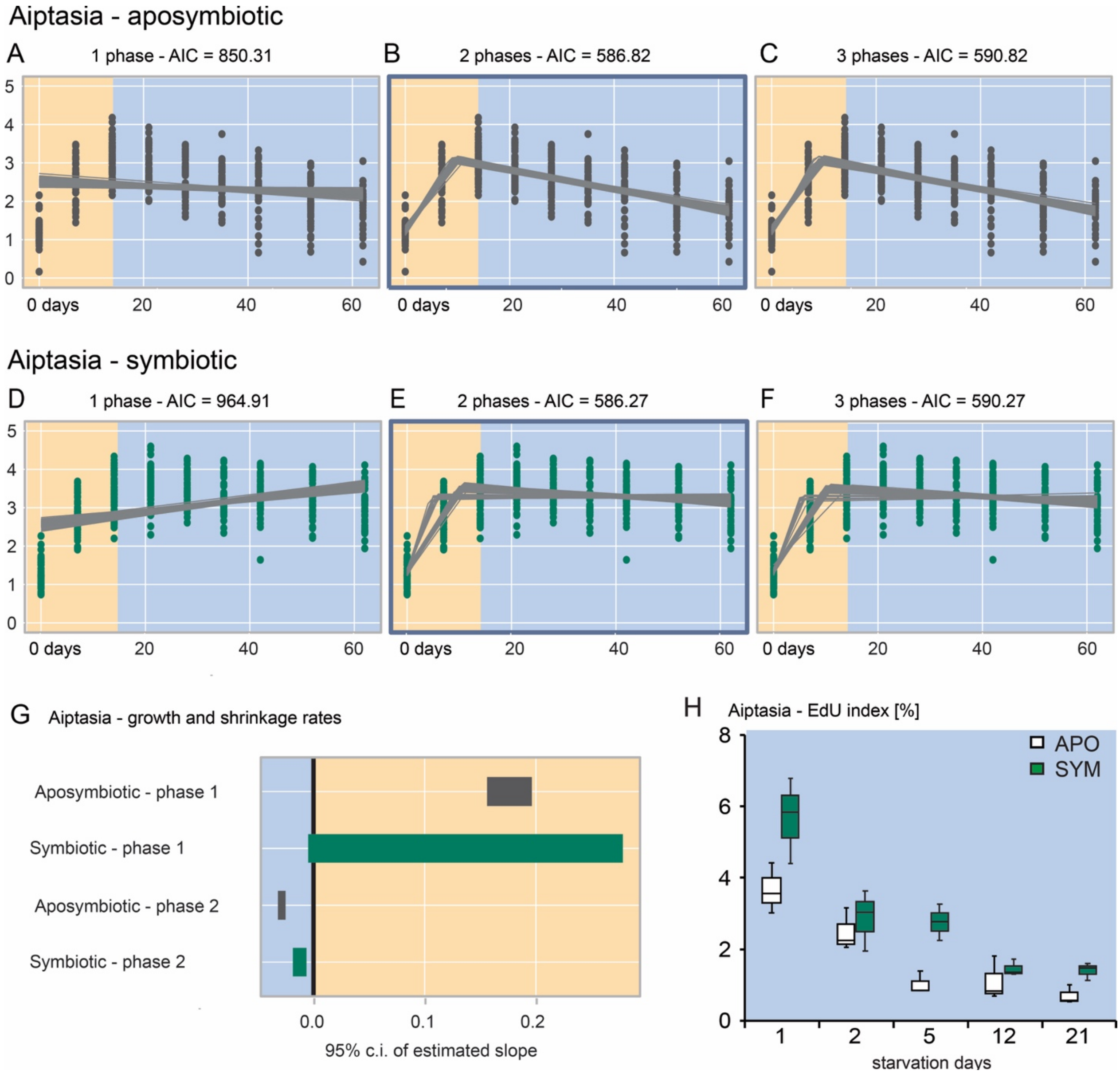

Fig. S5. Aiptasia polyps show feeding-dependent growth and shrinkage during starvation. (A-F) Multi-phased linear regression models were fitted to the log-transformed values of pedal disk area (see Fig. 1H) in aposymbiotic (A-C) and symbiotic (D-F) Aiptasia polyps. For both conditions, two phases best described the dynamics of size changes with a growth phase followed by a shrinkage phase. See SI for details on model selection. (G) Summary of slope values with 95% confidence interval for the best fitting change-point models. Note the overlap between growth slopes of symbiotic and aposymbiotic animals in 'phase 1'. During shrinkage in 'phase 2', shrinkage rates are higher in aposymbiotic than in symbiotic animals. (H) EdU index (60min pulse length) determined by flow cytometry at individual time points over the course of 21 days of starvation in aposymbiotic (APO; white) and symbiotic (SYM;

Development • Supplementary information

green) polyps of Aiptasia strain CC7. Two-way ANOVA reveals a significant effect of 'symbiosis state' ($F_{(1, 20)}$ = 20.498, p = 2.05E-04) and 'day' ($F_{(4, 20)}$ = 32.724, p = 1.62E-08) on the EdU index but no significant interaction effect between 'symbiosis state' and 'day' ($F_{(4, 20)}$ = 2.334, p = 0.091, see Table S3J). n=3 biological replicates of 3 individuals per time point.

Development • Supplementary information

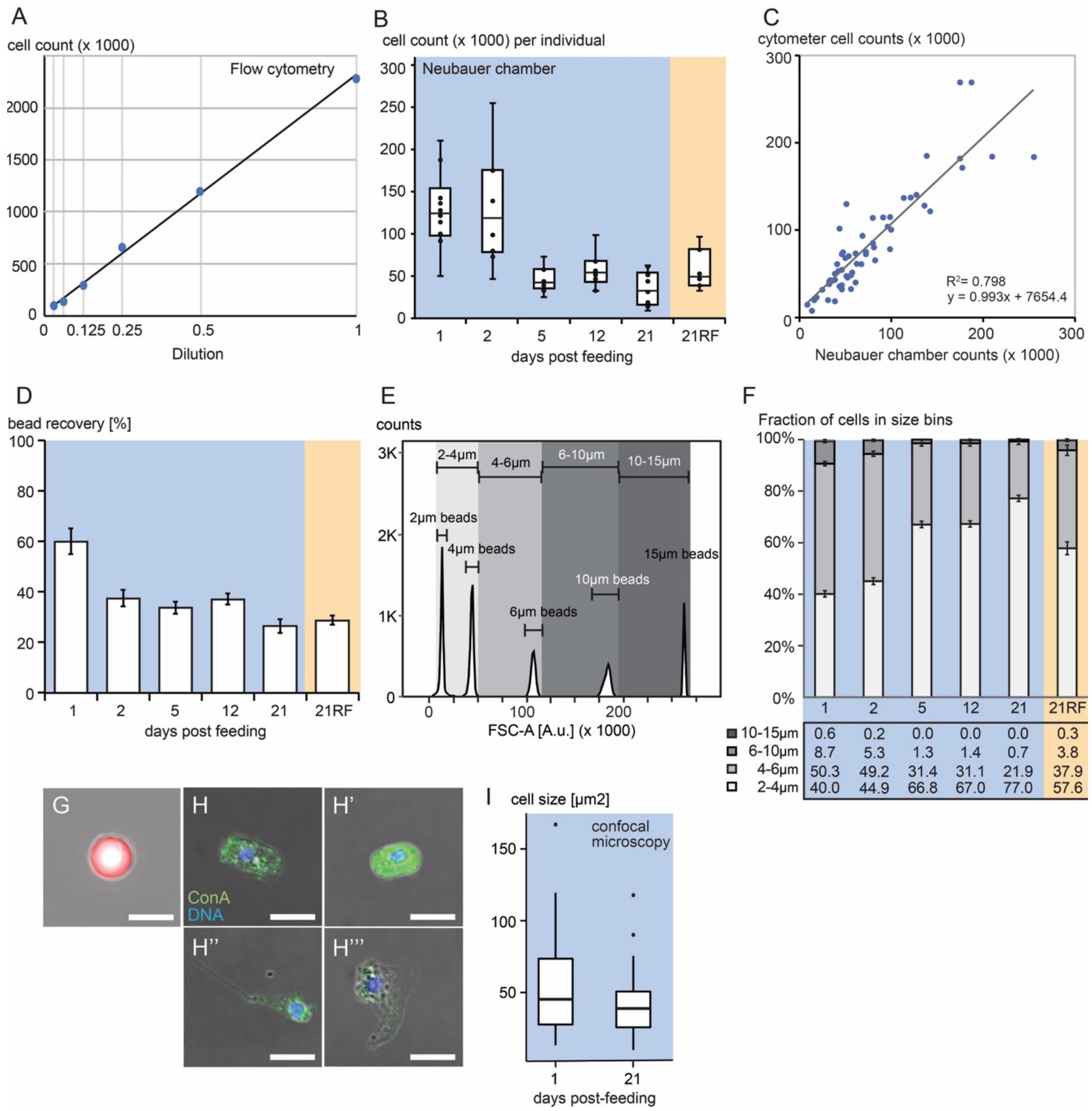

Fig. S6. Validation of flow cytometry estimates for individual cell size and cell number. (A) Serial dilutions of animal homogenates were used to benchmark the cell counts obtained by flow cytometry. We found a near-perfect correlation ($R^2 = 0.998$) between the recorded cell count and the dilution coefficient. Each datapoint represents the mean of 2 technical replicates from a dilution of cells originating from a pool of 10 dissociated individuals. (B, C) We validated cell numbers obtained by flow cytometry analysis (see Fig. 3G) in a subset of the flow cytometry samples (n=10 replicates per timepoint) by Neubauer chamber-supported manual cell counting (B). We found a good correlation between the methods (C, $R^2 = 0.798$). (D) Mean recovery rates of 10μm yellow-green FluoSpheres added before tissue homogenization to estimate cell loss during dissociation plus s.e.m. Note that bead recovery was calculated per

individual sample and used to correct the corresponding cell counts for bead/cell loss. (E) Overview of forward scatter (FSC-A) peaks of the size-standardized bead populations (ranging from 2–15μm diameter) used to correlate cytometric FSC-A values with cell size ranges. (F) The fraction of cells that overlap with different cell size bins during 21 days of starvation and after refeeding. Note that during starvation, the fraction of smallest cells (2-4 μm) increases at the expense of all other bins and that refeeding leads mainly to an increase of the 4-6 μm fraction at the expense of the 2-4 μm fraction. (G-H''') Example images of a 10 μm bead (G) or single cells (H-H''') from cell suspensions imaged by combined confocal and DIC microscopy to quantify average cell size. Green: Concanavalin-A cytoplasmic stain (ConA). Blue: FxCycle Violet nuclear stain. The size of 10 μm beads falls within the variance of cell sizes found in *Nematostella* (see Materials and Methods). (I) Cell size was approximated by measuring the average cell area at 1 or 21 days after feeding from confocal images, and showed a trend towards smaller cells in 21 days starved animals. Sample sizes (N_{d1}=42, N_{d21}=46) were likely too small to detect significant differences by microscopy (pairwise t-test, p = 0.174). A.u.: arbitrary units. s.e.m.: standard error of the mean.

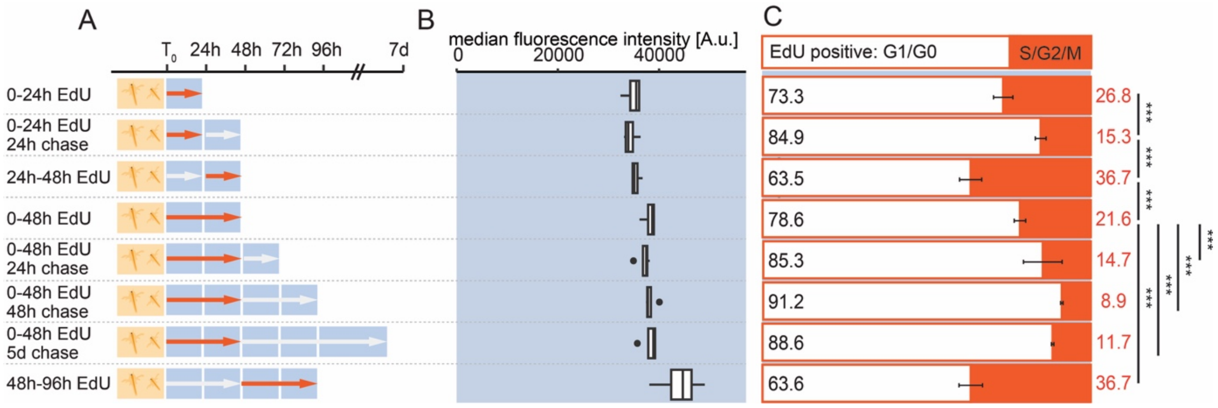

Fig. S7. Median fluorescent intensity measures of EdU label (B) and FxCycle violet DNA dye (C) points to a small population of cells proliferating feeding-independently. (A) Experimental setup of EdU pulse-chase experiments as in Fig. 4K-L. (B, C) Median EdU signal fluorescence intensity (B) and cell cycle distribution of EdU+ cells as estimated by EdU or FxCycle violet DNA dye signal intensity using flow cytometry (C). Statistical significance between pairwise comparisons was tested by Tukey's HSD post hoc test on one-way ANOVA analysis. ***= $p > 0.001$ (see Table S3G-I, Fig. S11 for gating strategy). Note that highest median EdU fluorescent intensity is observed during 48-96h EdU incubation, during which only a small number of cells is labelled (7.5±0.4% EdU+; Fig. 4L). These EdU+ cells also exhibit high proportions of cells in S/G_2/M indicating that they consist of a feeding-independent population of proliferating cells. Same samples as in Fig. 4K-L. A.u.: Arbitrary units.

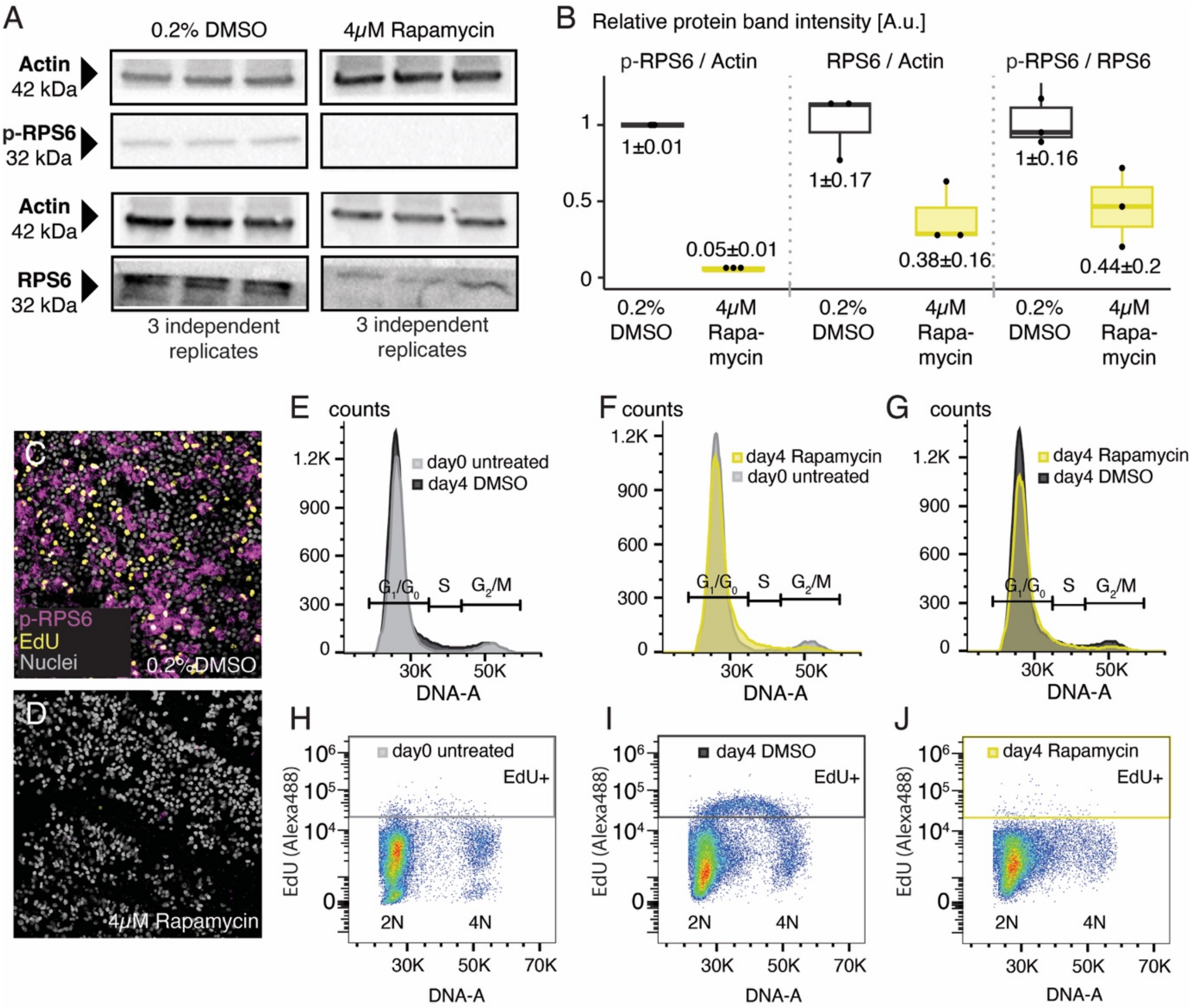

Fig. S8. Rapamycin treatment inhibits TOR signaling and changes cycle dynamics during feeding. (A) Western blots depict protein levels of Actin (as control), phosphorylated (p-RPS6) and unphosphorylated ribosomal protein S6 (RPS6) during feeding and 4 days of 0.2% DMSO or 4*µ*M Rapamycin. For each sample, protein from pools of 50 juveniles was extracted. (B) Relative levels of p-RPS6 or RPS6 band intensities show that TOR inhibition using 4µM Rapamycin led to a decrease of global RPS6 and of p-RPS6 levels. (C, D) Representative confocal imaging stacks of juvenile midbody epidermis after 4 days of feeding and 0.2% DMSO or 4 µM Rapamycin treatment. Note that p-RPS6 (purple) and EdU signal (yellow; 30 min EdU pulse) were nearly abolished in Rapamycin-treated animals. Nuclei stained by Hoechst33342. (E-J) Representative plots of flow cytometry analysis of EdU-labeled cell suspensions (30min EdU pulse) from 4-days starved juveniles at T_0 ('day0 untreated') and after 4 days of feeding and 0.2% DMSO, or 4*µ*M Rapamycin treatment (see also Fig. 5). Pools of 7-10 animals were dissociated for each of the biological replicates (n=3).The

comparison between starved, untreated T_0 and 4 days fed/0.2% DMSO controls (E, H-I) shows an increased proportion of S-phase cells (E) and of EdU+ cells as recognizable by the characteristic arch of EdU+ events connecting the 2N and 4N populations (compare H and I). Notably, Rapamycin led to an increased fraction of S-phase cells compared to T_0 (F), comparable to DMSO controls (G). However, only very few EdU+ events could be detected (J) and proportion of G_2/M phase cells is decreased after Rapamycin treatment (G). Together, this indicates that a proportion of cells gets arrested in S-phase without progressing to G_2/M. DNA-A: area of FxCycle™ Violet DNA dye.

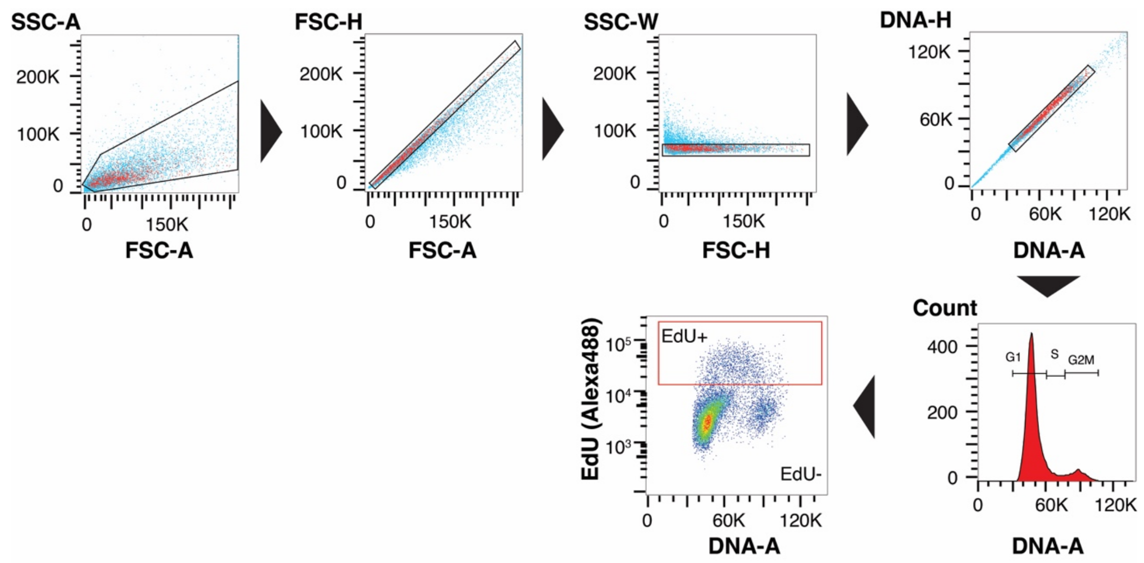

Fig. S9. Gating strategy in short EdU pulse (30-60min) experiments in *Nematostella* (refers to experiments in Fig. 4I, J, Fig. 5C-E). In *Nematostella* short pulse EdU experiments, 7 animals constituted one biological sample and 3 biological samples were performed for each time point. We coupled incorporated EdU with Alexa488 fluophore azide (recorded in the FITC cytometer channel). 1μg/ml FxCycle violet DNA dye (Invitrogen) was used to distinguish cell cycle phases (recorded in the "Pacific blue" cytometer channel 'DNA-A'). We first excluded debris based on size and granularity in the FSC-A/SSC-A gate, with sub-gates based on FSC-A/FSC-H parameters and FSC-A/SSC-W parameters to remove potential cell doublets and high complexity events. We then gated particles based on DNA dye intensity in width over area and plotted a histogram of DNA dye in area on the linear scale to visualize the characteristic DNA dye intensity peaks expected from cells between 2N and 4N. Based on the fluorescence signal of DMSO controls in the FITC-A channel within the 2N-4N pool of cells, a threshold was drawn above which cells were considered EdU+.

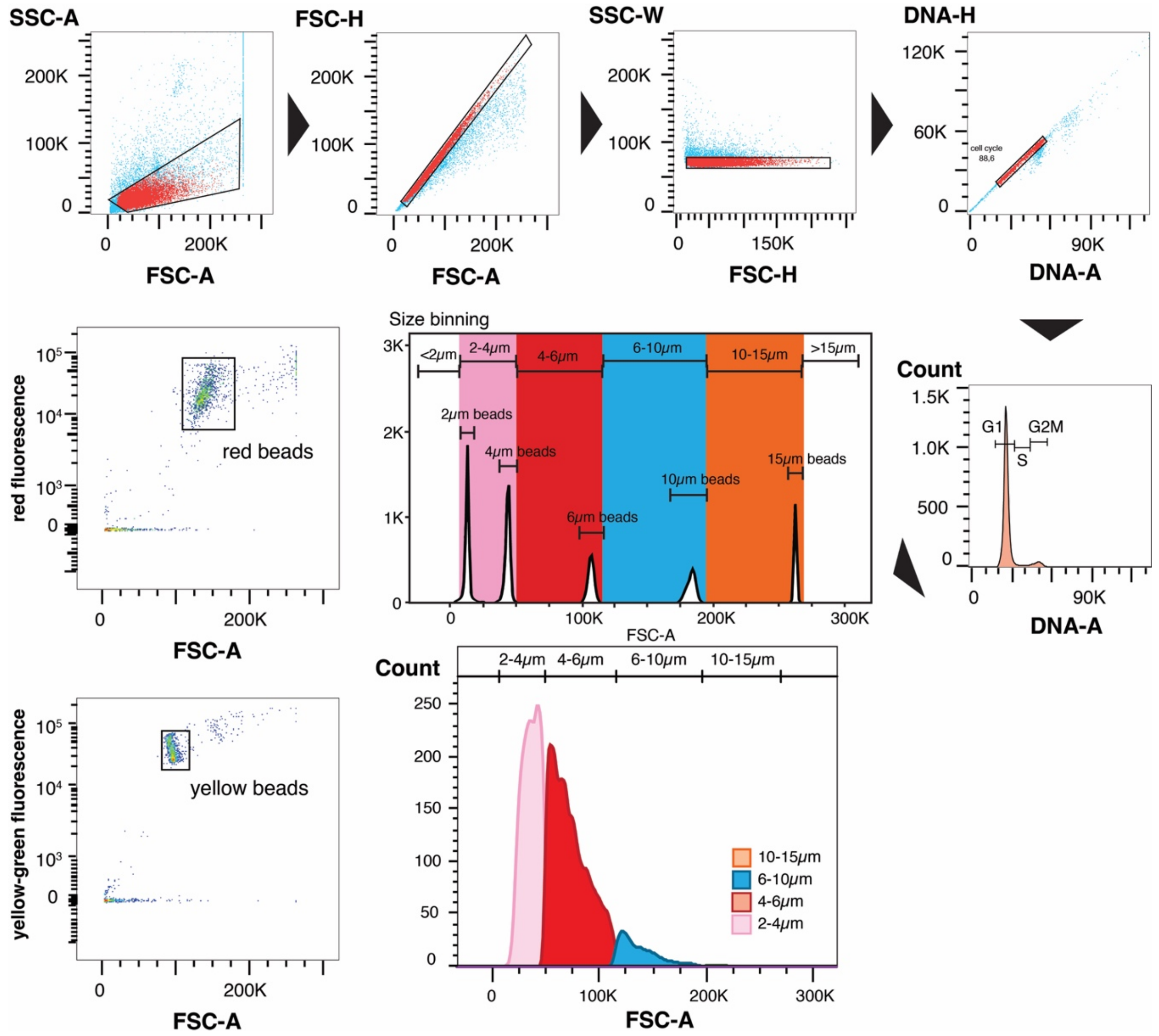

Fig. S10. Gating strategy to assess *Nematostella* cell numbers and cell sizes via cytometry (refers to experiments in Fig. 4J, S6F). In cell counting experiments, the number of cells per individual were estimated by the addition of a constant number of red fluorescent beads (10 µm FluoSpheres™ (580/605), Invitrogen; recorded in the PE channel). Based on the logic explained above, we excluded debris and gated cells within the cell cycle based on DNA-dye intensity. Using the ratio of counted beads to counted cells within the cell cycle allowed to back-calculate initial cell numbers per sample (see Materials and Methods). In the starvation experiment, a second set of yellow-green beads (10 µm FluoSpheres™ (505/515), Invitrogen; recorded in FITC channel) was added at a known concentration before dissociation and counted by cytometry to assess cell loss during dissociation. In addition, non-fluorescent polystyrene beads (with known diameter of 2.0 µm, 4.0 µm, 6.0 µm, 10.0 µm and 15.0 µm, Invitrogen) were used to create a reference of FSC-A values and allowed overlapping bead size-bins with the cells in the cell cycle gate to assess how cell size fractions change during starvation time points.

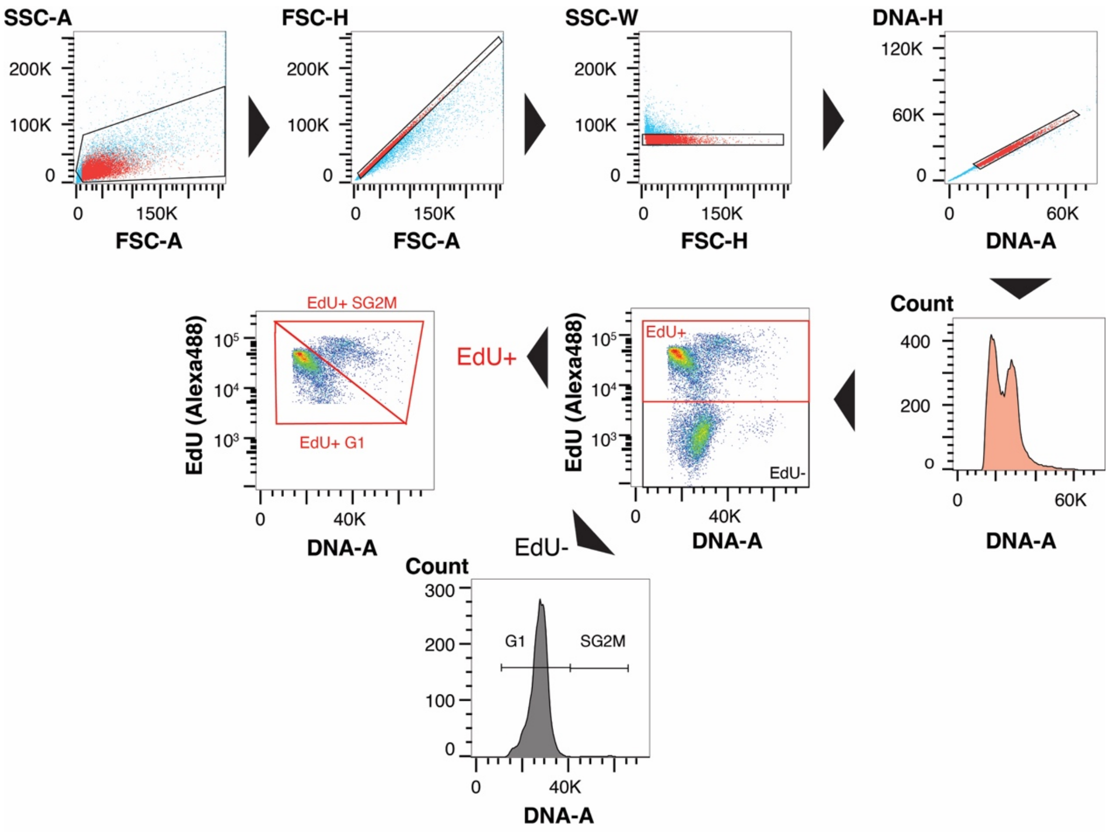

Fig. S11. Gating strategy in long (>24h) EdU pulse/chase experiments in *Nematostella* (refers to experiments in Fig. 4K, L, S7). For the analysis of a long (>24h) EdU pulse, we excluded debris and gated cells based on DNA-dye intensity as explained above. However, we observed that the long-term incorporation of EdU interfered with the DNA stain fluorescence and prevented a clear separation of 2N-4N cells. We therefore split the EdU+ from the EdU- cell populations based on a DMSO negative control and assessed the cell cycle separately. In the EdU+ cells, we differentiated a population of G_1/G_0 events, with lower EdU/DNA fluorescence and a population of $S/G_2/M$ events with higher EdU/DNA signal. The cell cycle distribution of EdU-negative cell populations was defined by gating from a DNA-signal histogram. As expected, the level of $S/G_2/M$ cells in the EdU-negative population after a long EdU pulse was negligible.

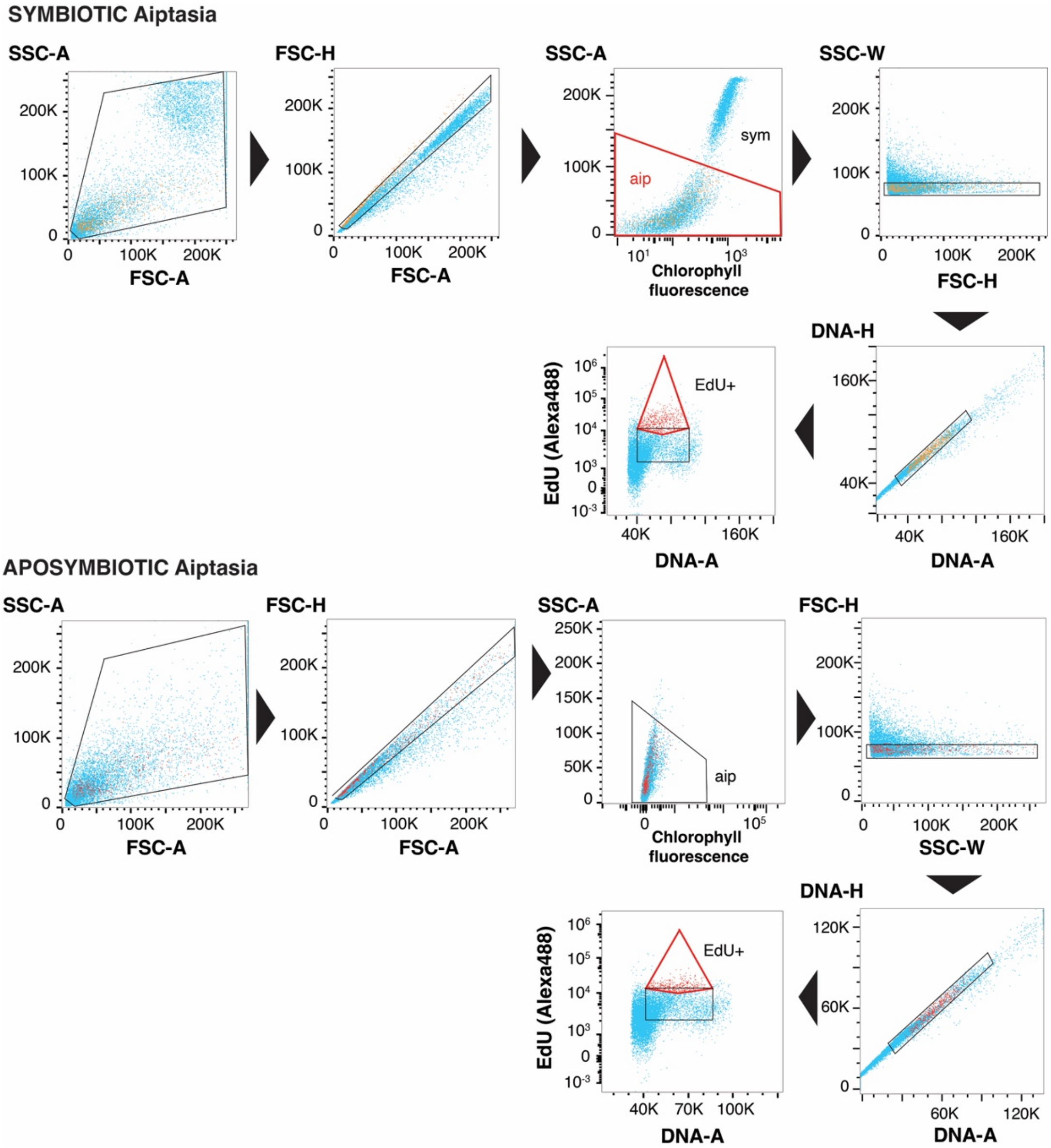

Fig. S12. Gating strategy in short EdU pulse (60min) experiments in Aiptasia (refers to experiments in Fig. S5H). In Aiptasia short pulse EdU experiments, 3 polyps of the strain CC7 in either aposymbiotic and symbiotic state, constituted one biological sample and 3 biological samples were performed for each time point. For visualizing incorporated EdU we used Alexa488 fluophore azide (recorded in the FITC cytometer channel) and stained DNA with FxCycle violet (Invitrogen). For both symbiotic and apo-symbiotic animals, chlorophyll autofluorescence (recorded in PE channel) was used to distinguish symbiont-free Aiptasia cells from symbionts/symbiont-containing Aiptasia cells. EdU+ cells were gated based on DMSO negative controls above in the DNA signal range of S-phase cells.

Table S1A. Slope estimates from multi-phase linear models for *Nematostella* and Aiptasia body size changes

Table S1B. Changepoints in multi-phased linear models for *Nematostella* and Aiptasia body size changes

Table S1C. Analysis of *Nematostella* growth / shrinkage rates in dependence of the individual starting size, phase (1-4) and feeding condition (RES vs AL)

Available for download at
https://journals.biologists.com/dev/article-lookup/doi/10.1242/dev.202926#supplementary-data

Table S2A. Summary of body size, cell number, cell size values during *Nematostella* growth and shrinkage

Table S2B. Changes in body size, cell number and cell size between timepoints

Table S2C. Linear regression analysis of individual bodysize, cell number and cell size

Table S2D. Linear model to estimate the effect of cell size or cell number on body size

Table S2E. Linear model to estimate the combined effect of cell size and cell number on body size

Table S2F. Pairwise comparisons (Tukey's HSD) between starvation timepoints based on an ANOVA to describe body size, cell number or cell size changes

Available for download at
https://journals.biologists.com/dev/article-lookup/doi/10.1242/dev.202926#supplementary-data

Table S3A. Fraction of EdU positive cells as counted by images from confocal microscopy

Table S3B. Flow cytometer analysis after 60min of EdU pulse - cell cycle phases defined as per DNA content

Table S3C. Comparison of the effect of the assay method (microscopy vs cytometry), day, and their interaction on the EdU index (ANOVA)

Table S3D: ANOVA for the effect of starvation day on the fraction of EdU positive cells and pairwise comparisons between days (Tukey's HSD)

Table S3E. ANOVA for the effect of starvation day on the fraction of EdU positive cells and pairwise comparisons between days (Tukey's HSD)

Table S3F. Flow cytometer analysis of long term EdU pulse (>24h) - cell cycle phases defined as per DNA content

Table S3G. ANOVA for the effect of experimental treatment on the fraction of EdU+ cells and pairwise comparisons between treatments (Tukey's HSD)

Table S3H. ANOVA for the effect of experimental treatment on the fraction of EdU+ S/G2M cells and pairwise comparisons between treatments (Tukey's HSD)

Table S3I. ANOVA for the effect of experimental treatment on the log (FITC-A) intensity as a quantification of EdU incorporation in EdU positive cells and pairwise comparisons between treatments (Tukey's HSD)

Table S3J. ANOVA for the effect of symbiotic state (symbiotic/aposymbiotic) on the fraction of EdU positive cells in Aiptasia and pairwise comparisons between treatments (Tukey's HSD)

Available for download at
https://journals.biologists.com/dev/article-lookup/doi/10.1242/dev.202926#supplementary-data

Development • Supplementary information

Table S4A. Bodysize before and after 4 days of daily feeding under DMSO (0.2%) or Rapamycin (4µM) treatment

Table S4B. EdU index before and after 4 days of daily feeding under DMSO (0.2%) or Rapamycin (4µM) treatment

Table S4C. Cell cycle composition before and after 4 days of daily feeding under DMSO (0.2%) or Rapamycin (4µM) treatment

Table S4D. Median FSC-A as a proxy for cell size before and after 4 days of daily feeding under DMSO (0.2%) or Rapamycin (4µM) treatment

Table S4E. ANOVA for the effect of day (day 0 vs day 4) and treatment (DMSO (0.2%) vs Rapamycin (4µM) on bodysize

Table S4F. ANOVA for the effect of DMSO (0.2%) or Rapamycin (4µM) treatment on the EdU index

Table S4G. ANOVA for the effect of DMSO (0.2%) or Rapamycin (4µM) treatment on the fraction of cells in S-phase

Table S4H. ANOVA for the effect of DMSO (0.2%) or Rapamycin (4µM) treatment on median FSC-A (as proxy for cell size)

Available for download at
https://journals.biologists.com/dev/article-lookup/doi/10.1242/dev.202926#supplementary-data

Supplementary Materials and Methods

1 Statistical methods

1.1 Software

Data preparation, statistical analysis and visualisations were performed using R [8], ggplot2 [11], ggpubr, and writexl [6]. Previous investigation used the R libraries lmtest [15], tidyverse [12], dplyr [13], gridExtra [1] and ggridges [14], and Python [9] with libraries matplotlib [4], numpy [3], pandas [7] and seaborn [10].

Data and code for the model fitting and statistics is freely available and can be accessed at `github.com/StochasticBiology/anemone-dynamics`.

1.2 Simple linear regressions for feeding and refeeding periods

Some of our data tracks body size over time in different experimental setups. Other data tracks body size, cell number, and cell size over time. For relationships between (a) a phenotypic variable and time or (b) two phenotypic variables that showed no evidence of changepoint behaviour, we used simple linear regression to explore the relationship between variables. For the phenotypic variables, we universally use log transformations and work with the transformed variable. Then, for variables x and y, the linear regression model is:

$$y = \beta_0 + \beta_1 x + \epsilon, \tag{1}$$

where β_1 is the slope of the relationship, β_0 the intercept, and $\epsilon \sim N(0, \sigma)$ is normally-distributed random noise with mean 0 standard deviation σ.

There are two main aspects that justify to work with the logarithmic transformation. First, we checked that it provides the property of homoscedasticity in the data, so that the variances of the observations y_i for $i = 1, \ldots, n$ are all very similar at each discrete time point (day), meeting an assumption for simple regression analysis. Secondly, by using the logarithmic transformation, we can fit linear regressions and then naturally interpret these models as exponential growths or decays in the original variables (body size, cell number or cell size).

For these fits, we report means and standard deviations of estimators for the slope β_1 and intercept β_0, as well as the coefficient of determination R^2 and Akaike Information Criterion (AIC) for use in comparing different model structures.

1.3 Multiple regression between phenotypic variables

To explore the relationship between body size, cell size, and cell number, we also use a linear regression picture. Let b denote body size, n cell number and s cell size. All these variables are dependent on time in days x, but we consider how they scale together over the measured range of times:

$$\log b = \beta_0 + \beta_1 \log n + \beta_2 \log s + \epsilon \tag{2}$$

We also explore the effect that cell number and cell size independently have on body size. For that, we fit two simple linear regressions of the form

$$\log b = \beta_{0,n} + \beta_{1,n} \log n + \epsilon_n \quad (3)$$
$$\log b = \beta_{0,s} + \beta_{1,s} \log s + \epsilon_s \quad (4)$$

1.4 Multi-phase models for starvation periods and cyclic periods

The changes in body size and at a cellular level are more complex to understand during starvation periods. This is because the animals undergo different phases of growth and degrowth when there is a prolonged lack of food. Therefore, simple regression models do not longer capture the biological processes as accurately as they do for body growth and cell growth and proliferation in feeding periods.

We approach this problem by defining multi-phase linear models, describing piecewise linear behaviours, with changepoints at particular times corresponding to changes in the slope of the relationship between response variable and time.

A multi-phase model for a number of N phases has $N-1$ changing-time-points. We impose that the first changing-time-point is $\tau_0 = 0$. We denote by $i = 1,\ldots,N$ the ith phase of the model, between the changing-time-point τ_{i-1} and τ_i. We define a function $l(t)$ that gives the phase corresponding to a given value t, that is, $l(t) = \max\{i \mid t \geq \tau_{i-1}\}$. Then, this model is a piecewise linear function defined by:

$$y(t) = \beta_0 + \beta_{l(t)}(t - \tau_{l(t)-1}) + \sum_{j=1}^{l(t)-1} \beta_j(\tau_j - \tau_{j-1}). \quad (5)$$

Equivalently, if we consider $i = 1\ldots,N-1$ and we assume that $\tau_0 = 0$ and $\tau_N = \infty$, we can write the multi-phase, piecewise linear model by:

$$y(t) = \begin{cases} \beta_0 + \beta_1 t & \text{for } 0 \leq t \leq \tau_1 \\ \beta_{i+1}(t-\tau_i) + y(\tau_i) & \text{for } \tau_i < t \leq \tau_{i+1} \end{cases} \quad (6)$$

The set of parameters of the model is $\vec{\theta} = (\beta_0, \beta_1, \beta_2, \ldots, \beta_N, \tau_1, \ldots, \tau_{N-1}, \sigma)$, where β_0 is the intercept (y at $t = 0$), b_i is the slope in phase $i > 0$, τ_i is the time at which phase i ends, and σ is the standard error of the normally distributed residuals (assuming homoscedasticity).

We infer parameter values $\vec{\theta}$ by maximising the log likelihood function $L(\vec{\theta}|y(t))$ for given data $y(t)$. We maximise the log likelihood function, for models involving 2-4 phases, using the function *optim* in R using the 'BGFS' quasi-Newton method [5], using an initial flat profile $\beta_0 = \bar{y}$, $\beta_i = 0$ for $i > 0$, determined by initial investigation to give the best optimisation performance. For more complex models with over 4 phases, we use a custom-written simulated annealing method in C++. Model selection is done by choosing the model with the number of phases that has lowest AIC value. To obtain uncertainties for the predictors and parameter estimates, we employ bootstrap resampling [2] 100 times for each dataset, to obtain a distribution of estimates for the slopes and changepoints. From the distributions, we obtain 95% confidence intervals as the values between the 2.5^{th} and the 97.5^{th} percentiles in the distribution. We report summaries of the distributions of parameter estimates, with a focus on the slopes and the changepoints τ.

Specifically, the log likelihood function $L(\vec{\theta}|y(t))$ of this model is then defined by:

$$L(\vec{\theta}|y(t)) = L_1(\vec{\theta}|y(t)) + \sum_{i=1}^{N-1} L_i(\vec{\theta}|y(t)) \quad (7)$$

Development • Supplementary information

where

$$
\begin{aligned}
L_1 &= \sum_{0 \le t_j \le \tau_1} \log(N(y(t_j)|\beta_0 + \beta_1 t_j, \sigma)) \\
L_i &= \sum_{\tau_i \le t_j \le \tau_{i+1}} \log(N(y(t_j)|\beta_{i+1}(t_j - \tau_i) + y(\tau_i), \sigma)) \text{ for } i > 1.
\end{aligned} \tag{8}
$$

In several cases, the general structure in these multi-phase models is a first phase of growth, with positive slope, a second phase of degrowth, with negative slope, and a third phase of growth in the case of cell size, as cells reduce their size until a threshold where they cannot become smaller, and from there they become bigger again.

2 Geometry

From the correlation between body size and cell number and cell size in growth during feeding periods, we can give insights in how cell proliferation and cell growth increase the body size by studying the geometry of the body.

Nematostella vectensis has a cylindrical body shape, its body size is measured as the area (in mm^2) of a rectangular projection of this cylinder into a viewing plane. For r denoting the radius of the base of the cylinder and h the height, the formulas of areas and volumes that we need are the following: area of a rectangle (body size)

$$A_{rectangle} = 2rh$$

which can change in width $2r$ or in height h. Area of a cell (cell size)

$$A_{cell} = \alpha,$$

volume of a cylinder:

$$V_{cylinder} = \pi r^2 h$$

which can change radially πr^2 or in elongation h, and lastly, volume of a cube with edge length l; $V_{cube} = l^3$.

The parameters of the correlation of body size and cell number and cell size during growth in feeding periods can be connected with this argument. With associated uncertainty, the slope for log body size with log cell number is approximately 2/3, and the slope for log body size with log cell size is approximately 1, hence a rough scaling law is:

$$b(s, n) \sim (s)^1 (n)^{2/3} \tag{9}$$

with b, n and s denoting body size, cell number and cell size, respectively.

As cell size, like body size, is measured as a 3D volume projected into an observed 2D plane, the linear scaling of observed body size with observed cell size intuitively corresponds to body size scaling like cell size. The 2/3 scaling with cell number suggests that increasing cell number may contribute sublinearly to either the length or radius, or both, of the 3D body form, which is unsurprising given the complexity of the body plan. Further detailed physiological work will dissect this relationship further.

Development • Supplementary information

3 Doubing times, half-lives and interval loss rates in starvation

We have generalyl observed that phenotypic variables in *Nematostella vectensis* undergo exponential growth and exponential decay of the form $N(t) = N_0 e^{rt}$ where r is the growth rate (if positive) or degrowth rate (if negative). These rates r of exponential behaviour are not necessarily the most interpretable way that this behaviour can be quantitatively reported. It is informative to compute the doubling times T_d and the half-lives $t_{1/2}$, giving the times that it takes for the initial quantity to double in number or to reduce to half, respectively. The corresponding expressions are, for doubling time,

$$T_d = \frac{\log(2)}{r}, \tag{10}$$

and for half-life,

$$t_{1/2} = \frac{\log(2)}{|r|}. \tag{11}$$

References

[1] Baptiste Auguie, Anton Antonov, and Maintainer Baptiste Auguie. Miscellaneous functions for "grid" graphics. *Repository CRAN*, Version 2.3, 2017.

[2] Daniel Berrar and Werner Dubitzky. *Bootstrapping*, pages 158–162. Springer New York, New York, NY, 2013. doi:10.1007/978-1-4419-9863-7_644.

[3] Charles R. Harris, K. Jarrod Millman, Stéfan J. van der Walt, Ralf Gommers, Pauli Virtanen, David Cournapeau, Eric Wieser, Julian Taylor, Sebastian Berg, Nathaniel J. Smith, Robert Kern, Matti Picus, Stephan Hoyer, Marten H. van Kerkwijk, Matthew Brett, Allan Haldane, Jaime Fernández del Río, Mark Wiebe, Pearu Peterson, Pierre Gérard-Marchant, Kevin Sheppard, Tyler Reddy, Warren Weckesser, Hameer Abbasi, Christoph Gohlke, and Travis E. Oliphant. Array programming with NumPy. *Nature*, 585(7825):357–362, September 2020. doi:10.1038/s41586-020-2649-2.

[4] J. D. Hunter. Matplotlib: A 2d graphics environment. *Computing in Science & Engineering*, 9(3):90–95, 2007. doi:10.1109/MCSE.2007.55.

[5] John C. Nash. *Compact numerical methods for computers: linear algebra and function minimisation*. Routledge, 2018.

[6] Jeroen Ooms. writexl: Export data frames to excel 'xlsx'format. *R package version*, 1, 2020.

[7] The pandas development team. pandas-dev/pandas: Pandas, February 2020. doi:10.5281/zenodo.3509134.

[8] RStudio Team. *RStudio: Integrated Development Environment for R*. RStudio, PBC., Boston, MA, 2020. URL: http://www.rstudio.com/.

[9] Guido Van Rossum and Fred L. Drake Jr. *Python reference manual*. Centrum voor Wiskunde en Informatica Amsterdam, 1995.

[10] Michael L. Waskom. seaborn: statistical data visualization. *Journal of Open Source Software*, 6(60):3021, 2021. doi:10.21105/joss.03021.

[11] Hadley Wickham. *ggplot2: Elegant Graphics for Data Analysis.* Springer-Verlag New York, 2016. URL: https://ggplot2.tidyverse.org.

[12] Hadley Wickham, Mara Averick, Jennifer Bryan, Winston Chang, Lucy D'Agostino McGowan, Romain François, Garrett Grolemund, Alex Hayes, Lionel Henry, Jim Hester, Max Kuhn, Thomas Lin Pedersen, Evan Miller, Stephan Milton Bache, Kirill Müller, Jeroen Ooms, David Robinson, Dana Paige Seidel, Vitalie Spinu, Kohske Takahashi, Davis Vaughan, Claus Wilke, Kara Woo, and Hiroaki Yutani. Welcome to the tidyverse. *Journal of Open Source Software*, 4(43):1686, 2019. doi:10.21105/joss.01686.

[13] Hadley Wickham, Romain François, Lionel Henry, Kirill Müller, and Davis Vaughan. *dplyr: A Grammar of Data Manipulation*, 2023. https://dplyr.tidyverse.org, https://github.com/tidyverse/dplyr.

[14] Claus O. Wilke. *ggridges: Ridgeline Plots in 'ggplot2'*, 2022. R package version 0.5.4. URL: https://wilkelab.org/ggridges/.

[15] Achim Zeileis and Torsten Hothorn. Diagnostic checking in regression relationships. *R News*, 2(3):7–10, 2002. URL: https://CRAN.R-project.org/doc/Rnews/.
